# Deciphering anti-infectious compounds from Peruvian medicinal *Cordoncillos* extract library through multiplexed assays and chemical profiling

**DOI:** 10.3389/fphar.2023.1100542

**Published:** 2023-06-05

**Authors:** Pedro G. Vásquez-Ocmín, Sandrine Cojean, Vincent Roumy, Guillaume Marti, Sébastien Pomel, Alice Gadea, Karine Leblanc, Indira Dennemont, Liliana Ruiz-Vásquez, Hivelli Ricopa Cotrina, Wilfredo Ruiz Mesia, Stéphane Bertani, Lastenia Ruiz Mesia, Alexandre Maciuk

**Affiliations:** ^1^ UMR152 PHARMADEV, IRD, UPS, Université de Toulouse, Toulouse, France; ^2^ Université Paris-Saclay, CNRS, BioCIS, Orsay, France; ^3^ CNR Du Paludisme, AP-HP, Hôpital Bichat–Claude Bernard, Paris, France; ^4^ Joint Research Unit 1158 BioEcoAgro, University Lille, JUNIA, INRAE, University Liège, UPJV, University Artois, ULCO, VilleneuveD’Ascq, France; ^5^ Laboratoire de Recherche en Sciences Végétales (UMR 5546), CNRS, Université de Toulouse, Toulouse, France; ^6^ MetaboHUB, National Infrastructure of Metabolomics and Fluxomics, Toulouse, France; ^7^ Facultad de Farmacia y Bioquímica, Universidad Nacional de la Amazonía Peruana (UNAP), Iquitos, Peru; ^8^ Centro de Investigación de Recursos Naturales, Universidad Nacional de la Amazonía Peruana (UNAP), Iquitos, Peru; ^9^ Facultad de Ingeniería Química, Universidad Nacional de la Amazonía Peruana (UNAP), Iquitos, Peru; ^10^ International Joint Laboratory of Molecular Anthropological Oncology (LOAM), National Cancer Institute, Lima, Perú

**Keywords:** anti-infectious diseases, metabolomics, Peruvian amazonia, *Piper*, Cordoncillos

## Abstract

High prevalence of parasitic or bacterial infectious diseases in some world areas is due to multiple reasons, including a lack of an appropriate health policy, challenging logistics and poverty. The support to research and development of new medicines to fight infectious diseases is one of the sustainable development goals promoted by World Health Organization (WHO). In this sense, the traditional medicinal knowledge substantiated by ethnopharmacology is a valuable starting point for drug discovery. This work aims at the scientific validation of the traditional use of *Piper* species (“*Cordoncillos*”) as firsthand anti-infectious medicines. For this purpose, we adapted a computational statistical model to correlate the LCMS chemical profiles of 54 extracts from 19 *Piper* species to their corresponding anti-infectious assay results based on 37 microbial or parasites strains. We mainly identified two groups of bioactive compounds (called features as they are considered at the analytical level and are not formally isolated). Group 1 is composed of 11 features being highly correlated to an inhibiting activity on 21 bacteria (principally Gram-positive strains), one fungus (*C. albicans*), and one parasite (*Trypanosoma brucei gambiense*). The group 2 is composed of 9 features having a clear selectivity on *Leishmania* (all strains, both axenic and intramacrophagic). Bioactive features in group 1 were identified principally in the extracts of *Piper strigosum* and *P. xanthostachyum*. In group 2, bioactive features were distributed in the extracts of 14 *Piper* species. This multiplexed approach provided a broad picture of the metabolome as well as a map of compounds putatively associated to bioactivity. To our knowledge, the implementation of this type of metabolomics tools aimed at identifying bioactive compounds has not been used so far.

## Introduction

In Amazonia, tropical diseases have a high prevalence due to multiple reasons, including a lack of an appropriate health policy, a climate conducive to diseases, challenging logistics and poverty. Neglected tropical diseases (NTDs) are a group of 20 conditions (caused by viruses, protozoa, helminths and bacteria) prioritized by the World Health Organization (WHO). The NTDs are responsible for approximately 200,000 deaths and the loss of 19 million disability-adjusted life years (DALYs) annually. In 2020, new infections by *Plasmodium* spp. and *Leishmania* spp. worldwide were estimated at 241 and 1 million, respectively. Even if *Plasmodium* is not strictly classified as a NTD, it caused around 627,000 deaths in 2020, with two-thirds of these deaths (470,000) being due to treatment disruptions during the COVID-19 pandemic (WHO, 2021). *Leishmania* spp. and *Trypanosoma* spp. are protozoan parasites responsible for diseases labeled as NTDs *stricto sensu*. The ambitious 10-year WHO plan to defeat NTDs is based on three pillars: control, elimination and eradication (WHO, 2022a). The support of research and development of new medicines to fight infectious diseases is one of the sustainable development goals promoted by WHO (WHO, 2022b). Drug resistance is a widespread concern in medical care, and the increase of drug-resistant infections is faster than the pace of the development of new drugs approved for use in humans. Therefore, every input into the search for new antimicrobial agents is welcome (Yan et al., 2021). Ethnopharmacology is the interdisciplinary study of the knowledge or practices of traditional cultures related to plants, animals, or mineral used for therapeutic purposes (SFE, 2022). Such knowledge can be valued as the starting point for drug discovery, especially in the case of infectious diseases which are prevalent among such populations.

Piperaceae is a pantropical family composed of eight genera. Two genera are the most representative in this family: *Piper* and *Peperomia* (Vásquez-Ocmín et al., 2017; Salehi et al., 2019). Among these, *Piper* is the most diverse and representative genus, encompassing *ca.* 2600 species (Trujillo et al., 2022). Besides pungent compounds, many *Piper* species produce essential oils and are hence highly aromatic, explaining their use for cooking and medicinal purposes (Ruiz-Vásquez et al., 2022). In Peru, *Piper* spp. (called “Cordoncillos”) have been used for a very long time in traditional medicine as a “first-hand treatment”, especially in the villages far away from major cities and medical care. The necessary scientific validation of traditional uses implies the isolation of the main bioactive compounds by successive fractionations using chromatographic techniques and biological activity testing (bio-guided isolation). Such an approach has evidenced interesting activities for *Piper* spp. as anti-inflammatory, antiparasitic, antibacterial, *etc.* (Mgbeahuruike et al., 2017; Durant-Archibold et al., 2018). Even if bio-guided fractionation is still used with some success in the natural product chemistry field, current trends involve streamlining the cost, effort, and time (Vásquez-Ocmín et al., 2022). Metabolomics is a holistic approach allowing rapid detection and putative identification (*i.e.*, annotation) of numerous metabolites, along with data mining on multiple datasets. Metabolomics aims to comprehensively map all biochemical reactions in a given system and has become a key to deciphering their biological roles, hence becoming mainstream in natural product chemistry and drug discovery. This approach can be divided into targeted and untargeted analyses (Alarcon-Barrera et al., 2022). Two spectral techniques are regularly employed for metabolomics analysis: mass spectrometry (MS) and nuclear magnetic resonance (NMR) spectroscopy, both being assisted by bioinformatics and statistical analysis. Our team has solid expertise in implementing straightforward and effective workflows to decipher anti-infectious compounds using untargeted metabolomics (Vásquez-Ocmín et al., 2021a).

This work aims at the scientific validation of the use of *Piper* species as firsthand anti-infectious medicines. We adapted a statistical model to correlate the LCMS chemical profiles of 54 extracts from 19 *Piper* species to their corresponding anti-infectious assay results based on 37 microbial or parasites strains. This multiplexed approach led to the annotation of compounds bearing these activities.

## Materials and methods

### Ethnopharmacology and plant material

Based on the encouraging previous results of our research group on the anti-infectious activities of *Piper* species (Vásquez-Ocmín et al., 2021a), ethnopharmacological surveys were realized as part of the project “Compuestos bioactivos *in vitro* a partir de especies vegetales Amazónicas”. The surveys were undertaken between July and December 2020 in communities of three Amazonian regions of Peru: Cusco, Loreto and San Martin. People of these communities were interrogated about the main use of medicinal *Piper* species (“*Cordoncillos*”), including their use against malaria, “uta” (local name for leishmaniasis) and bacteria. According to ethnopharmacological studies, we collected 8 species in Cusco, 10 species in Loreto, and 1 species in San Martin.

Thus, different parts of these nineteen plants were collected, then identified and deposited in the Herbarium Amazonense (AMAZ), Iquitos, Peru. This project was realized in accordance with the guidelines pertaining to ethnopharmacological studies and edited by the Laboratorio de Investigacion de Productos Naturales Antiparasitarios de la Amazonia (LIPNAA) of the Universidad Nacional de la Amazonia Peruana (UNAP) (Resolucion Rectoral Nº 1312-2020-UNAP).

### Preparation of plant extracts

One hundred grams of each air-dried and ground plant (leaves, leaves and stems, aerial parts) were soaked in 1 L of each of the following solvents successively: hexane, methylene chloride, methanol, ethanol/water (7:3, v/v), and aqueous. These macerations were performed during 21 days each for hexane, methylene chloride and ethanol/water, and for 15 days in methanol and aqueous extracts, with solvents being changed every 3 days. The extracts were then filtered through a paper filter and evaporated under reduced pressure below 40 °C. Dry extracts were stored at −4 °C until use. Extracts were solubilized in DMSO at a concentration of 10 mg/mL for *in vitro* bioassays and HPLC-MS analysis.

### Cell lines and microorganism culture

*HUVEC cells:* Human umbilical vein endothelial cells (HUVECs) were maintained in culture in RPMI 1640 medium (Invitrogen, Life Technologies) supplemented with 10% heat-inactivated fetal bovine serum (Invitrogen Life Technologies) and 1 mM glutamine (Invitrogen Life Technologies) (Vásquez-Ocmín et al., 2018).

*RAW264.7:* The mouse monocyte/macrophage cell line RAW264.7 was maintained in culture in DMEM (Invitrogen, Life Technologies) supplemented with 10% heat-inactivated fetal bovine serum (Vásquez-Ocmín et al., 2018).

*Plasmodium*: The *P. falciparum* chloroquine-sensitive strain 3D7 was obtained from the Malaria French National Reference Center (CNR Paludisme, Hôpital Bichat Claude Bernard, Paris) and was maintained in O^+^ human erythrocytes in RPMI 1640 medium (Invitrogen, Life Technologies) supplemented with 25 mM HEPES (Sigma), 25 mM NaHCO_3_ (Sigma) and 0.5% Albumax II (Invitrogen, Life Technologies) at 37 °C in a candle-jar method following the Trager and Jensen conditions (Trager and Jensen, 1976; Lambros and Vanderberg, 1979; Vásquez-Ocmín et al., 2018)**.**

*Leishmania*: The *Leishmania donovani* MHOM/ET/67/HU3, *L. amazonensis* MHOM/BR/73/M2269, and *L. braziliensis* MHOM/BR/75/M2903 b strains were maintained routinely in *vitro* culture. Passages in macrophages RAW 264.7 were carried out regularly to preserve the virulence of the strain, then they were recovered for culture maintenance in the promastigote form. For assays, parasites were maintained as promastigote forms in M-199 medium (Sigma) supplemented with 40 mM HEPES, 100 mM adenosine, 0.5 mg/L hemin and 10% fetal bovine serum (FBS) at 25 °C in a dark environment (Balaraman et al., 2015; Vásquez-Ocmín et al., 2018).

*Trypanosomes*: Trypomastigotes of *T b. gambiense* (FéoITMAP/1893 strain) were grown in HMI9 medium constituted of prepacked Iscove’s modified Dulbecco’s medium (Thermo-Fisher, Les Ulis, France) supplemented with 36 mM NaHCO_3_, 1 mM hypoxanthine, 0.05 mM bathocuproine, 0.16 mM thymidine, 0.2 mM 2-mercapthoethanol, 1.5 mM L-cysteine, 10% heat-inactivated foetal bovine serum, 100 IU penicillin and 100 μg mL^-1^ streptomycin. Parasites were incubated in a Series 8,000 direct-heat CO_2_ incubator (Thermo-Fisher, Les Ulis, France) at 37 °C in a water-saturated atmosphere containing 5% CO_2_ (Pomel et al., 2015; Vásquez-Ocmín et al., 2018).

*Bacteria and yeast*: Most microbial strains were diluted in BH medium (Brain Heart), MH medium (Mueller Hinton) for *Candida sp*. and *Mycobacterium* sp., or WW medium (Wilkins-West) for *Streptococcus* sp. and stored at −20 °C. Then strains were subcultured at 20 °C on RC (Ringer Cysteine) medium for 24 h before tests (Bocquet et al., 2019).

### *In vitro* antiprotozoal activity

#### *In vitro* antiplasmodial activity on *P. falciparum*

Assays were realized with a suspension of erythrocytes at 1% parasitemia containing more than 85% ring stage obtained by repeated sorbitol treatment and incubated with the compounds at concentrations ranging between 0.49 and 100 µM or µg/mL, obtained by serial dilution, in duplicates. Two controls were used, parasites without drug and parasites with chloroquine at concentrations ranging between 0.49 and 1000 nM. Plates were incubated for 44 h at 37 °C in a candle jar (Vásquez-Ocmín et al., 2018; 2021a).

#### *In vitro* antileishmanial activity on *L. donovani*, *L. amazonensis*, and *L. braziliensis* axenic amastigotes

A suspension of promastigotes in growth plateau-phase was incubated at 37 °C in 5% CO_2_ for 3 days to obtain the amastigote form in promastigote medium supplemented with 2 mM CaCl2, 2 mM MgCl_2_, and a pH adjusted to 5.5. The axenic amastigote suspension containing 1.106 parasites/mL was incubated for 72 h with the compounds at 37 °C in 5% CO_2_ in the dark. Tested compounds or extracts were obtained by serial dilution and ranged between 0.49 and 100 µM or µg/mL. There were two controls: parasites without drug and parasites treated with miltefosine at the same concentrations as the compounds tested (Vásquez-Ocmín et al., 2018; 2021a).

#### *In vitro* antileishmanial activity on *L. donovani*, *L. amazonenesis* and *L. braziliensis* intramacrophage amastigote form

Macrophages were seeded into a 96 well microtitration plate at a density of 100,000 cells/well in 100 μL and incubated in a 5% CO_2_ at 37 °C for 24 h. After removing the medium, cells were incubated with 100 μL of fresh DMEM containing a suspension of promastigotes in the growth plateau phase at a rate of 1 cell per 10 parasites. After incubation under a 5% CO_2_ atmosphere at 37 °C for 24 h (the time needed by the parasite to infect the macrophage), the culture medium was replaced with 100 μL of fresh DMEM with different concentrations of compounds as previously for a new incubation period of 48 h. Controls were parasites alone in DMEM medium, axenic amastigotes, macrophages alone, infected macrophages and infected macrophages with different concentrations of miltefosine (Balaraman et al., 2015; Vásquez-Ocmín et al., 2018; 2021a).

#### Determination of IC_50_, CC_50_ and selectivity index for *Plasmodium*, *Leishmania*, and *Trypanosoma*

After incubation, the plates were subjected to 3 freeze/thaw cycles to achieve complete cell lysis. The cell lysis suspension was diluted 1:1 except for Plasmodium plates that have been diluted 1:10 in lysis buffer (10 mM NaCl, 1 mM Tris HCl pH 8, 2.5 mM EDTA pH 8, 0.05% SDS, 0.01 mg/mL proteinase K and 1X SYBR Green). SYBR Green incorporation in cell DNA amplification was determined using the Master epRealplex cycler^®^ (Eppendorf, France) and the following program to increase SYBR Green incorporation: 90 °C for 1 min, decrease in temperature from 90 °C to 10 °C for 5 min with fluorescence reading at 10 °C for 1 min and a new reading at 10 °C for 2 min. Molecules were tested in duplicate. Compounds with different SYBR Green fluorescence values from duplicates were retested. The cytotoxicity of the compounds was expressed as IC_50_ (concentration of drug inhibiting the parasite growth by 50%, comparatively to the controls), CC_50_ (Cytotoxic Concentration 50%: concentration inhibiting macrophages growth by 50%). IC_50_ and CC_50_ were calculated by nonlinear regression using ICEstimator 1.2 version (http://www.antimalarial-icestimator.net/MethodIntro.htm). Selectivity index (SI) for antiplasmodial and anti-*Trypanosoma* activities were calculated as the HUVEC’s CC_50_ divided to IC_50_ of *Plasmodium* 3D7 and *Trypanosoma*, and for *Leishmania* assays as the ratio of RAW 364.7 CC_50_ to intramacrophage amastigote IC_50_ value (Vásquez-Ocmín et al., 2018; 2021a).

#### *In vitro* evaluation on bloodstream form of *Trypanosoma brucei gambiense*

For each extract, twelve two-fold serial dilutions from 100 μg/mL to 0.049 μg/mL were performed in 96-well microplates in 100 µL HMI9 medium. Parasites were then added to each well to reach a final density of 4.104 cells/mL in 200 µL. Following 3 days of incubation at 37 °C with 5% CO_2_ in a water-saturated atmosphere, 20 µL of resazurin at 450 µM in water was added to each well for a final concentration of 40.9 nM to evaluate cell viability. Plates were then incubated for 6 h at 37 °C with 5% CO_2_ in water-saturated atmosphere and the conversion of resazurin to resorufin was quantified by measuring the absorbance at 570 nm (resofurin) and 600 nm (resazurin) using the microplate reader Spark^®^ (Lyon, France) Pentamidine di-isethionate was used as the reference compound (Pomel et al., 2015; Vásquez-Ocmín et al., 2018; 2021a).

#### Antimicrobial assay

Minimal Inhibitory Concentration (MIC) determinations of crude extracts were carried out using the agar dilution method stipulated by the Clinical and Laboratory Standards Institute (CLSI, 2006). Antimicrobial activity was evaluated for the first time against a panel of 36 pathogenic and multi-drug-resistant bacteria, which, in most cases, have been recently isolated from human infections. For comparison, reference strains from the American Type Culture Collection (ATCC) were included.

The inhibitory concentrations ranged between 0.075 and 1.2 mg/mL in five dilutions (1.2, 0.6, 0.3, 0.15 and 0.075 mg/mL); 0.075 mg/mL was considered a low enough concentration for a preliminary screening. Petri dishes were inoculated with strains (104 CFU, obtained by dilution in brain heart medium, BH) using a Steer’s replicator and were incubated at 37°C for 24 h. MIC was defined as the lowest concentration of extract without bacterial growth after incubation. The extracts with MIC ≤1.2 mg/mL were tested in triplicate at lower concentrations (mean absolute deviation is done for values: 1.2 ± 0.4; 0.6 ± 0.2; 0.3 ± 0.1; 0.15 ± 0.05; 0.075 ± 0.03). The standards (gentamicin, vancomycin, amoxicillin, amphotericin B, fluconazole, and sertaconazole) were tested in triplicate in 12 concentrations ranging from 0.03 to 64 mg/mL.

#### Cytotoxic assay

Cytotoxicity was evaluated on RAW 264.7 macrophages for *Leishmania* assays and HUVECs for *Plasmodium* and *Trypanosoma*. The cells were seeded at a density of 50,000 cells per well in 100 µl of DMEM in a 96-well microtiter plate. After incubation in a 5% CO_2_ at 37 °C for 24h, the culture medium was replaced with 100 µl of fresh DMEM containing serial dilutions of the compounds tested. The concentrations of the compounds are the same as for intramacrophage *Leishmania* or *Plasmodium* assays. The plates were incubated for 48 h at 37 °C with 5% CO_2_. Antiprotozoal assays have been performed only with compounds not demonstrating cytotoxicity (Vásquez-Ocmín et al., 2021a).

### Liquid chromatography and mass spectrometry data mining

#### HPLC-MS analyses

Extracts were analyzed by liquid chromatography performed on an Agilent 1260 series HPLC coupled to a 6530 QToF (Agilent Technologies). Chromatography separations were performed on a XSelect CSH C18 column, 130Å, 2.5 µm, 2.1 × 75 mm –(Waters). The mobile phase comprised water (0.1% formic acid) A) and acetonitrile (ACN) B). A stepwise gradient method at a constant flow rate of 0.35 mL/min was applied as follows: 5%–100% B (0–9.5 min), 100% B (4.5 min) and 4 min equilibration at 5% B. The mass spectrometer settings were: positive ESI mode, 50-3200 mass range calibration, and 2 GHz acquisition rate. Ionization source conditions were drying gas temperature 325 °C, drying gas flow rate 10 L/min, nebulizer 35 psig, fragmentor 150 V, and skimmer 65 V. Range of *m/z* was 200–1700. Purine C_5_H_4_N_4_ [M + H]^+^ ion (*m/z* 121.050873) and the hexakis-(1H,1H, 3H-tetrafluoropropoxy)-phosphazene C_18_H_18_F_24_N_3_O_6_P_3_ [M + H]^+^ ion (*m/z* 922.009798) were used as internal lock masses. Full scans were acquired at a resolution of 11,000 (at m/z 922). MS-MS acquisitions were performed using three collision energies: 10, 20, and 40 eV. Three of the most intense ions (top 3) per cycle were selected. MS-MS acquisition parameters were defined as follows: *m/z* range 100–1200, default charge of 1, minimum intensity of 5,000 counts, rate/time = 3 spectra/s, isolation width: Narrow (1.3 u).

#### Data processing

For untargeted metabolomics, the LC-MS data were processed according to the MSCleanR workflow (Fraisier-Vannier et al., 2020; Vásquez-Ocmín et al., 2021b). Briefly, a batch in positive ionization (PI) was processed with MS-DIAL version 4.90 (Tsugawa et al., 2015). MS1 and MS2 tolerances were set to 0.01 and 0.05 Da, respectively, in centroid mode for each dataset. Peaks were aligned on a QC reference with an RT tolerance of 0.2 min, a mass tolerance of 0.015 Da, and a minimum peak height detection at 1 × 10^5^. MS-DIAL data was deconvoluted together with MS-CleanR by selecting all filters with a minimum blank ratio set to 0.8 and a maximum relative standard deviation (RSD) set to 40. The maximum mass difference for feature relationship detection was set to 0.005 Da, and the maximum RT difference was set to 0.025 min. Pearson correlation links were used with a correlation ≥0.8 and a *p*-value significance threshold of 0.05. Two peaks were kept in each cluster for further database requests and the kept features were annotated with MS-FINDER version 3.52 (Tsugawa et al., 2016). The MS1 and MS2 tolerances were set to 5 and 15 ppm, respectively. The formula finder was exclusively based on C, H, O, and N atoms. Three levels of compounds annotation from several sets of data were carried out. Metabolite annotation at level 1 using MS-DIAL: i) the experimental LC-MS/MS data of 500 compounds (retention time, exact mass and fragmentation) were used as references; ii) the Mass spectral records from MS-DIAL, MONA (MassBank of North America), and GNPS (Global Natural Product Social Molecular Networking) databases were used for spectral match applying a dot product score cut-off of 800. Metabolite annotation at level 2 was prioritized according to: i) a search would be made using MSFinder for a match with compounds identified in the literature for the *Piper* genus (genus level) and Piperaceae family (family level) (Dictionary of Natural Products version 28.2, CRC Press) based on exact mass and *in silico* fragmentation. For metabolite annotation level 3, a search was made using MS-FINDER for a match with natural compounds included in their databases embedded (PlantCyc, ChEBI, NANPDB, COCONUT, and KNApSAcK) (generic level) (Vásquez-Ocmín et al., 2023).

Mass spectra similarity networking was carried out from PI mode using MetGem (Olivon et al., 2018) on the final annotated. msp file and metadata file for PI obtained with MSCleanR. Values used were MS2 m/z tolerance = 0.02 Da, minimum matched peaks = 4 and minimal cosine score value = 0.7. The visualization of the molecular network (MN) was performed on Cytoscape version 3.9.1 (Shannon et al., 2003). The list of compound mass, retention time, row ID and peak height was exported in CSV format. Then, this format was used for the statistical data analysis.

##### Statistical analyses

The final annotated untargeted metabolome feature matrix and biological assay results datasets were analyzed using the R package MixOmics (http://mixomics.org/), which is dedicated to the integrative analysis of ‘omics’ data (Le Cao et al., 2016). Biological assays results expressed in IC_50_ were transformed in pIC_50_ (-log10 [IC_50_]) for further statistical correlation analysis. MIC values were log transformed. The LC-MS dataset (*m/z* × RT × peak area) was normalized to total ion chromatogram and scaled to unit variance. A vertical integration approach has been afforded to leverage on multiplexed measurements for the same extract (González et al., 2008). To highlight the overall correlation between chemical fingerprints and biological assays. A regularized Canonical Correlation Analysis (rCCA) was done using a cross validation approach for tuning regularization parameters. The correlation structure of the two-block data matrices was displayed on clustered image map (CIM) and relevance network using 0.3 as cutoff correlation value.

## Results

### Ethnopharmacological survey

Our set of 19 samples was collected in three Amazonian regions of Peru, where the use of these species of “*Cordoncillos*” in traditional medicine is widespread. All information about the collected samples are presented in Supplementary Table S1. Among the plants studied in this work, 10 (52.6%) had not been subject to chemical analysis so far. In our previous work (Vásquez-Ocmín et al., 2021a), *Piper casapiense*, *P. strigosum* and *P. pseudoarboreum* showed promising antiprotozoal activity. Thus, these plants were re-collected in other areas of the Loreto region.

### Anti-infectious activity

#### Antiprotozoal activity

The IC_50_ values for antiplasmodial, antileishmanial and antitrypanosomal activities of each extract are shown in Table 1. We considered an extract active and worthy to be further studied when its IC_50_ value was below 10 μg/ml. Four extracts were active in this case regarding the 3D7 *P. falciparum* chloroquine-sensitive strain assay: the hexane and methylene chloride extracts of *P. crassinervium*, the methanol extract of *P. stellipilum*, and the methylene chloride extract of *P. xanthostachyum*. Only the methylene chloride extracts of *P. crassinervium*, and *P. oblongum* were active on three strains of *Leishmania* (both axenic and intra-macrophagic amastigotes). For the other active extracts, six were not active on intra-macrophagic forms: hexane and methylene chloride of *P. heterophyllum*, hexane, methylene chloride and water extracts of *P. sancti-felicis* (*L. amazonensis*) and a hexane extract of *P. crassinervium* (*L. donovani*). Hexane extract of *P. “cordatomentosa”* was active on all the strains, both axenic and intramacrophagic amastigotes, except on axenic amastigote forms of *L. donovani*. Five extracts were active only on *L. amazonenesis*, and *L. braziliensis* (both axenic and intramacrophagic amastigotes): methylene chloride extracts of *P. calvescentinerve*, *Piper crassinervium*, and *P. oblongum*, and the methylene chloride and hexane extracts of *Piper “cordatomentosa”, and P. crassinervium*. Five extracts were active only on *L. donovani*, and *L. braziliensis* (both axenic and intramacrophagic amastigotes), the methylene chloride extract of *P. crassinervium*, and the hexane and methylene chloride extracts of *P. heterophyllum*, and *P. oblongum*. Hexane extract of *P. glabribaccum* was not active on axenic amastigote forms of *L. donovani*. Two extracts were active on *L. donovani*, and *L. amazonenesis*, both forms, methylene chloride extracts of *P. crassinervium*, and *P. oblongum*. Selective activity on *L. braziliensis* were observed for twelve extracts, the hexane and methylene chloride extracts *Piper sancti-felicis*, *Piper stellipilum*, and *P. xanthostachyum*; hexane and methylene chloride extracts of *P. stellipilum*, and hexane and methylene chloride extracts of *P. calvescentinerve* and of *P. divaricatum*. None of the extracts showed selective activity on *L. donovani* or *L. amazonensis*.

**TABLE 1:** *In vitro* antiprotozoal and cytotoxicity activities of *Piper* extracts. M = methanol; H = hexane; D = methylene chloride (D stands for dichloromethane); HA = hydroalcoholic; Aq = aqueous; NT = not tested. *Species showing no reference in phytochemical literature on Web of Science and PubMed. **Extracts previously tested (Vásquez-Ocmín et al., 2021a) (the plants in this work come from different collection 331 site). L = leaves; L&S = Leaves and stems; AP = aerial parts; “” = species with temporary botanical name (these species need more taxonomical 332 studies).

*Species, Region* (part tested)	Extract (code for metabolomic analyses)	*P. falciparum* 3D7 strain, IC_50_± SD, µg/mL	*L. donovani* LV9 strain (axenic amastigotes), IC_50_± SD, µg/mL	*L. donovani* LV9 strain (intra-macrophagic amastigotes), IC_50_± SD, µg/mL	*L. amazonensis* (axenic amastigotes), IC_50_± SD, µg/mL	*L. amazonensis* (intra-macrophagic amastigotes), IC_50_± SD, µg/mL	*L. braziliensis* (axenic amastigotes), IC_50_± SD, µg/mL	*L. braziliensis* (intra-macrophagic amastigotes), IC_50_± SD, µg/mL	*Trypanosoma brucei gambiense* IC_50_± SD, µg/mL	HUVEC CC_50_± SD, µg/mL	RAW 264.7 macrophages CC_50_± SD, µg/mL
*Piper casapiense* (Miq.) C. DC., Loreto (L&S)	H (P4H)	>100	>100	NT	>100	NT	>100	NT	0.68 ± 0.14	>100	>100
	D (P4D)**	>100	>100	NT	>100	NT	>100	NT	4.06 ± 1.11	45.31 ± 1.97	61.12 ± 4.41
	M (P4M)	>100	>100	NT	60.64 ± 6.07	44.22 ± 3.3	>100	NT	18.59 ± 11.62	30.48 ± 2.55	64.88 ± 6.98
*Piper strigosum* Trel.; Loreto (L&S)	H (P12H)**	>100	>100	NT	74.24 ± 6.33	22.41 ± 3.12	17.41 ± 0.71	9.07 ± 0.22	0.10 ± 0.02	>100	>100
	D (P12D)**	>100	>100	NT	54.43 ± 4.22	31.12 ± 2.66	12.33 ± 0.43	8.12 ± 0.87	0.028 ± 0.004	41.66 ± 4.01	66.01 ± 5.13
	M (P12M)**	>100	>100	NT	54.21 ± 4.32	20.97 ± 2.08	20.22 ± 0.20	17.64 ± 1.64	4.22 ± 1.59	51.22 ± 4.20	>100
*Piper pseudoarboreum* Yunck; Loreto (L&S)	H (P14H)**	>100	>100	NT	>100	NT	>100	NT	12.06 ± 3.80	61.94 ± 4.64	>100
	D (P14D)**	>100	>100	NT	>100	NT	>100	NT	5.07 ± 2.17	40.21 ± 4.12	>100
	M (P14M)**	>100	>100	NT	>100	NT	>100	NT	28.56 ± 15.97	25.49 ± 1.87	49.09 ± 3.88
*Piper armatum Trel. & Yunck; Loreto (L&S)	H (P18H)	>100	>100	NT	>100	NT	>100	NT	25.04 ± 5.33	>100	>100
	D (P18D)	>100	>100	NT	>100	NT	>100	NT	1.50 ± 0.16	55.31 ± 4.12	>100
	M (P18M)	>100	>100	NT	>100	NT	>100	NT	11.26 ± 1.90	35.86 ± 2.88	33.03 ± 2.99
*Piper brasiliense C. DC; Loreto (L&S)	H (P19H)	>100	>100	NT	23.45 ± 2.74	20.12 ± 1.66	14.20 ± 1.33	12.66 ± 1.64	29.47 ± 12.31	>100	>100
	D (P19D)	20.19 ± 1.44	>100	NT	12.66 ± 1.64	10.22 ± 1.99	18.41 ± 1.84	11.22 ± 1.88	1.11 ± 0.13	20.19 ± 1.44	21.99 ± 2.37
* “Piper bullatum Vahl”; Cusco (L&S)	H (P20H)	>100	>100	NT	>100	NT	>100	NT	5.05 ± 1.51	>100	77.64 ± 6.88
	D (P20D)	>100	>100	NT	>100	NT	>100	NT	4.40 ± 0.54	43.79 ± 4.41	41.66 ± 3.64
	M (P20M)	>100	>100	NT	>100	NT	>100	NT	21.47 ± 12.60	25.09 ± 2.40	>100
*Piper calvescentinerve Trel; Cusco (L&S)	H (P21H)	>100	29.29 ± 2.12	22.64 ± 2.01	17.44 ± 1.55	15.84 ± 1.11	6.33 ± 0.63	1.92 ± 0.07	3.97 ± 0.25	>100	>100
	D (P21D)	>100	>100	NT	2.55 ± 1.08	1.94 ± 0.84	3.54 ± 0.16	1.64 ± 1.02	6.08 ± 0.49	38.83 ± 5.41	44.88 ± 4.34
	M (P21M)	>100	>100	NT	>100	NT	43.61 ± 3.87	20.64 ± 1.55	17.75 ± 1.32	38.55 ± 1.13	72.22 ± 6.66
*Piper “cordatomentosa”;* Cuzco (L&S)	H (P22H)	>100	12.41 ± 0.88	9.78 ± 1.55	8.77 ± 1.12	5.66 ± 1.88	6.92 ± 0.71	3.22 ± 0.48	4.50 ± 1.27	>100	>100
	D (P22D)	>100	17.99 ± 0.77	18.34 ± 0.66	10.44 ± 1.45	10.44 ± 1.84	4.59 ± 0.45	1.08 ± 0.33	2.62 ± 1.11	58.32 ± 5.68	61.44 ± 4.11
	M (P22M)	>100	>100	NT	>100	NT	>100	NT	34.58 ± 13.10	61.50 ± 2.62	>100
*Piper crassinervium* Kunth.; Cusco (L&S)	H (P23H)	5.66 ± 0.87	8.05 ± 0.74	>100	8.55 ± 1.61	4.55 ± 1.09	6.48 ± 1.22	3.18 ± 1.08	7.08 ± 0.85	3.51 ± 0.71	10.44 ± 0.79
	D (P23D)	7.41 ± 0.99	2.95 ± 0.32	0.99 ± 1.33	3.18 ± 1.08	2.66 ± 1.88	5.77 ± 1.12	1.25 ± 1.46	3.25 ± 0.62	>100	37.38 ± 4.52
	M (P23M)	>100	>100	NT	>100	NT	2.70 ± 0.19	1.12 ± 1.46	14.44 ± 9.07	15.28 ± 3.07	37.38 ± 4.52
*Piper divaricatum* G. Mey; San Martin L)	H (P24H)	>100	>100	NT	>100	NT	>100	NT	4.57 ± 0.94	30.04 ± 4.95	55.95 ± 4.12
	D (P24D)	>100	>100	NT	>100	NT	2.61 ± 0.27	5.44 ± 0.88	4.02 ± 0.39	>100	>100
*Piper glabribaccum Trel; Cusco (L&S)	H (P25H)	>100	12.05 ± 3.25	8.33 ± 1.48	12.44 ± 0.77	11.41 ± 1.61	3.58 ± 0.45	3.88 ± 0.44	4.83 ± 1.36	>100	>100
	D (P25D)	>100	5.04 ± 1.2	10.33 ± 2.10	13.41 ± 0.45	11.22 ± 0.43	3.57 ± 0.45	6.66 ± 0.64	3.85 ± 1.26	35.62 ± 2.54	77.20 ± 6.61
	M (P25M)	>100	>100	NT	>100	NT	>100	NT	14.98 ± 2.75	84.72 ± 1.25	>100
*Piper heterophyllum* Ruiz & Pav.; Loreto L)	H (P26H)	>100	4.32 ± 0.76	3.11 ± 0.99	15.66 ± 1.46	11.51 ± 0.98	5.55 ± 0.95	3.15 ± 0.99	17.27 ± 0.59	81.67 ± 2.17	89.66 ± 5.78
	D (P26D)	>100	6.56 ± 0.61	7.0 ± 0.61	10.41 ± 0.64	11.32 ± 1.61	2.07 ± 0.20	3.44 ± 0.88	11.59 ± 3.93	17.14 ± 2.19	50.53 ± 5.11
*Piper oblongum Kunth; Cusco (L&S)	H (P27H)	>100	8.02 ± 1.16	7.66 ± 1.11	13.45 ± 1.99	15.64 ± 1.66	5.03 ± 1.39	4.13 ± 1.22	4.91 ± 0.75	48.32 ± 1.18	51.64 ± 4.30
	D (P27D)	>100	3.70 ± 0.71	1.36 ± 0.88	8.77 ± 1.65	7.44 ± 0.78	3.58 ± 0.43	4.13 ± 0.99	5.94 ± 1.88	26.23 ± 1.01	>100
	M (P27M)	>100	>100	NT	>100	NT	5.03 ± 1.39	5.03 ± 1.40	10.65 ± 2.21	21.43 ± 1.99	77.56 ± 4.81
*Piper reticulatum* L; Loreto L)	H (P28H)	>100	>100	NT	>100	NT	>100	NT	11.84 ± 1.29	>100	>100
	D (P28D)	>100	>100	NT	>100	NT	>100	NT	6.65 ± 1.31	>100	84.46 ± 5.88
	M (P28M)	>100	>100	NT	>100	NT	>100	NT	24.92 ± 6.60	>100	77.54 ± 7.12
*Piper sancti-felicis Trel; Loreto (L&S)	H (P29 H)	>100	17.88 ± 1.11	18.41 ± 1.33	21.55 ± 1.46	18.22 ± 1.62	4.10 ± 0.57	3.44 ± 0.99	6.60 ± 1.24	>100	>100
	D (P29 D)	>100	13.11 ± 0.88	10.45 ± 0.46	18.41 ± 1.34	17.51 ± 1.99	4.21 ± 0.52	6.44 ± 0.99	9.23 ± 2.58	49.40 ± 4.04	>100
	Aq (P29 Aq)	>100	4.63 ± 0.63	1.12 ± 0.77	8.64 ± 0.44	11.51 ± 1.41	6.07 ± 0.16	10.31 ± 0.11	48.13 ± 8.38	72.22 ± 4.98	>100
*Piper stellipilum (Miq.) C. DC; Loreto (L&S)	H (P30H)	>100	>100	NT	>100	NT	3.74 ± 0.65	8.41 ± 1.31	6.78 ± 3.78	>100	>100
	D (P30D)	13.44 ± 0.21	>100	NT	>100	NT	4.45 ± 0.45	9.99 ± 1.34	9.64 ± 0.46	18.05 ± 1.04	66.54 ± 5.12
	M (P30M)	8.22 ± 0.77	>100	NT	>100	NT	12.33 ± 1.08	15.64 ± 1.88	13.15 ± 2.01	25.03 ± 3.09	51.44 ± 2.99
*Piper trigonum C. DC.; Cusco (L&S)	H (P31H)	>100	>100	NT	>100	NT	>100	NT	8.84 ± 0.29	>100	>100
	D (P31D)	>100	>100	NT	>100	NT	>100	NT	4.84 ± 1.40	30.51 ± 3.05	77.45 ± 4.44
	M (P31M)	>100	>100	NT	>100	NT	>100	NT	10.93 ± 2.53	>100	70.67 ± 6.12
*Piper verruculosum C. DC.; Cusco (L&S)	H (P32H)	>100	>100	NT	>100	NT	>100	NT	8.54 ± 2.90	>100	>100
	D (P32D)	>100	>100	NT	>100	NT	>100	NT	5.64 ± 0.41	>100	>100
	M (P32M)	>100	>100	NT	>100	NT	>100	NT	27.38 ± 6.81	>100	>100
*Piper xanthostachyum* C.DC.; Loreto L)	H (P33H)	>100	17.54 ± 0.99	11.12 ± 1.01	21.46 ± 1.89	18.44 ± 1.46	6.51 ± 0.64	9.55 ± 0.55	14.00 ± 1.26	>100	>100
	D (P33D)	6.33 ± 0.74	14.79 ± 0.77	15.32 ± 0.99	29.44 ± 1.46	15.63 ± 1.77	2.98 ± 0.29	5.45 ± 0.66	2.25 ± 0.83	6.18 ± 1.74	52.12 ± 4.03
	HA (P33HA)	>100	23.11 ± 1.08	18.47 ± 1.12	30.48 ± 2.66	15.66 ± 1.63	23.88 ± 0.89	21.41 ± 1.48	4.11 ± 0.69	49.84 ± 4.31	45.89 ± 4.08
Chloroquine		0.007 (21.40 ±	NT	NT	NT	NT	NT	NT	NT	NT	NT
		1.56 nM									
Miltefosine		NT	1.41 ± 0.50 (3.46 ± 1.22 μM)	2.49 ± 0.45 (6.10 ± 1.10 μM)	1.88 ± 1.39 (4.61 ± 3.41 μM)	2.15 ± 1.78 (5.28 ± 4.37 μM)	3.49 ± 1.85 (8.56 ± 4.54 μM)	2.22 ± 1.64 (5.44 ± 4.02 μM)	NT	NT	NT
Pentamidine		NT	NT	NT	NT	NT	NT	NT	0.006 ± 0.002 (17.6 ± 5.8 nM)	NT	NT

Selectivity index (SI) values for antiprotozoal activities (Table 2) were ranked as low >10 < 50, high >51 < 99, and very high >100. For antimalarial activity, the only active extract displaying a low SI (13.50) was the methylene chloride extract of *P. crassinervium*. For the antileishmanial activity, methylene chloride extract of *P. crassinervium* and hexane extracts of *Piper glabribaccum* and *P. heterophyllum* showed a low SI on *Leishmania donovani* with 37.76, 12.0, and 28.82 respectively; methylene chloride and aqueous extracts of *P. oblongum* and *P. sancti-felicis* showed high SI with 73.53 and 89.29 respectively. For *L. amazonenesis*, three extracts presented a low SI, methylene chloride extracts of *P. “cordatomentosa”*, *P. crassinervium*, and *P. oblongum* with 13.39, 14.05, and 13.44 respectively. For *L. braziliensis*, 17 extracts displayed low SI, the hexane extracts of *P. strigosum*, *P. “cordatomentosa”*, *P. glabribaccum*, *P. heterophyllum*, *P. oblongum*, *P. sancti-felicis*, *P. stellipilum*, and *P. xanthostachyum* (11.02, 31.06, 25.77, 28.46, 12.50, 29.07, 11.89, 10.47), methylene chloride extracts of *Piper calvescentinerve*, *P. crassinervium*, *P. divaricatum*, *P. glabribaccum*, *P. heterophyllum*, *P. oblongum*, and *P. sancti-felicis* (27.37, 29.90, 18.38, 11.59, 14.69, 24.21, 15.53, respectively); methanol extracts of *Piper crassinervium*, and *P. oblongum* (33.36 and 15.42). Only the hexane and methylene chloride extracts of *P. calvescentinerve* and *P. “cordatomentosa”* presented a high SI with 52.08 and 56.89, respectively.

**TABLE 2:** Selectivity index of *Piper* extracts calculated with CC_50_ on HUVEC cells for *P. falciparum* and *T. b. gambiense*, and CC_50_ on RAW 264.7 cells for *Leishmania* strains. M = methanol; H = hexane; D = methylene chloride (D stands for dichloromethane); Aq = aqueous; ND = not determined. *Species with no phytochemical reference in Web of Science and PubMed. **Extracts previously tested (Vásquez-Ocmín et al., 2021a) (the plants come from different collection site). L = leaves; L&S = Leaves and stems. “” = species with a temporary botanical name (these species need further taxonomic study).

*Species*	Extract (code for metabolomic analyses)	SI on *P. falciparum* 3D7 strain, CC_50_/IC_50_	SI on *L. donovani* (intra-macrophagic amastigotes), CC_50_/IC_50_	SI on *L. amazonenesis* (intra-macrophagic amastigotes), CC_50_/IC_50_	SI on *L. braziliensis* (intra-macrophagic amastigotes), CC_50_/IC_50_	SI on *T b. gambiense*, CC_50_/IC_50_
*Piper casapiense*	H (P4H)	≤1	ND	ND	ND	147.06
	D (P4D)**	0.45	ND	ND	ND	11.16
	M (P4M)	0.30	ND	1.46	ND	1.64
*Piper strigosum*	H (P12H)**	≤1	ND	4.46	11.02	1000
	D (P12D)**	0.42	ND	2.12	8.13	1487.86
	M (P12M)**	0.51	ND	4.77	5.67	12.14
*Piper pseudoarboreum*	H (P14H)**	0.62	ND	ND	ND	5.14
	D (P14D)**	0.40	ND	ND	ND	7.93
	M (P14M)**	0.25	ND	ND	ND	0.89
*Piper armatum	H (P18H)	≤1	ND	ND	ND	3.99
	D (P18D)	0.55	ND	ND	ND	36.87
	M (P18M)	0.36	ND	ND	ND	11.26
*Piper brasiliense	H (P19H)	≤1	ND	4.97	7.90	3.39
	D (P19D)	1.00	ND	2.15	1.96	18.19
*“Piper bullatum”	H (P20H)	≤1	ND	ND	ND	19.80
	D (P20D)	0.44	ND	ND	ND	9.95
	M (P20M)	0.25	ND	ND	ND	1.17
*Piper calvescentinerve*	H (P21H)	≤1	4.42	6.31	52.08	25.19
	D (P21D)	0.39	ND	2.31	27.37	6.39
	M (P21M)	0.39	ND	ND	ND	2.17
*“Piper cordatomentosa"*	H (P22H)	≤1	10.22	1.77	31.06	22.22
	D (P22D)	0.58	3.35	13.39	56.89	22.26
	M (P22M)	0.62	ND	ND	ND	1.79
*Piper crassinervium* Kunth	H (P23H)	0.62	0.10	2.99	3.28	0.50
	D (P23D)	13.50	37.76	14.05	29.90	30.77
	M (P23M)	0.15	ND	ND	33.36	10.58
*Piper divaricatum*	H (P24H)	0.30	ND	ND	ND	6.57
	D (P24D)	≤1	ND	ND	18.38	24.88
*Piper glabribaccum	H (P25H)	≤1	12.00	8.76	25.77	20.70
	D (P25D)	0.36	7.47	0.15	11.59	9.25
	M (P25M)	0.85	ND	ND	ND	5.66
*Piper heterophyllum*	H (P26H)	0.82	28.82	7.79	28.46	4.73
	D (P26D)	0.17	7.22	4.46	14.69	1.48
*Piper oblongum	H (P27H)	0.48	6.74	3.30	12.50	9.84
	D (P27D)	0.26	73.53	13.44	24.21	4.42
	M (P27M)	0.21	ND	ND	15.42	2.01
*Piper reticulatum*	H (P28H)	≤1	ND	ND	ND	8.45
	D (P28D)	≤1	ND	ND	ND	15.04
	M (P28M)	≤1	ND	ND	ND	4.01
*Piper sancti-felicis	H (P29 H)	≤1	5.13	5.49	29.07	15.15
	D (P29 D)	0.49	9.57	5.71	15.53	5.35
	Aq (P29 Aq)	0.72	89.29	8.69	9.70	1.50
*Piper stellipilum	H (P30H)	≤1	ND	ND	11.89	14.75
	D (P30D)	1.34	ND	ND	6.66	1.87
	M (P30M)	3.04	ND	ND	3.29	1.90
*Piper trigonum	H (P31H)	≤1	ND	ND	ND	11.31
	D (P31D)	0.31	ND	ND	ND	6.30
	M (P31M)	≤1	ND	ND	ND	9.15
*Piper verruculosum*	H (P32H)	≤1	ND	ND	ND	11.70
	D (P32D)	≤1	ND	ND	ND	17.73
	M (P32M)	≤1	ND	ND	ND	3.65
*Piper xanthostachyum*	H (P33H)	≤1	8.99	5.42	10.47	7.14
	D (P33D)	0.98	3.40	3.33	9.56	2.75
	HA (P33HA)	0.50	2.48	2.93	2.14	12.13

At least one extract of all nineteen plants, except *P. heterophyllum*, was active on *T b. gambiense* at IC_50_ ≤ 10 μg/mL (Table 1). The only two plants that displayed activity in all extracts were *P. strigosum* and *P. divaricatum*. Among all active extracts, five had very good activity with a IC_50_ ≤ 1 μg/mL, hexane and methylene chloride extracts of *P. strigosum*, hexane extract of *P. casapiense*, and methylene chloride extracts of *P. armatum*, and *P. brasiliense*. Especially, the methylene chloride extract from *Piper strigosum* was very active with an IC_50_ at 0.028 μg/mL. Three extracts presented a high SI, hexane extract of *P. casapiense* with 147.06 and the hexane and methylene chloride extracts of *P. strigosum* with 1000 and 1487.86, respectively. Twenty extracts displayed a lower yet interesting SI between 10.58 and 36.87.

#### Antimicrobial activity

Antimicrobial assay results are presented in (Supplementary Table S2) (17 Gram-positive bacteria, 13 Gram-negative bacteria, 2 yeasts). Extracts were more active on Gram-positive bacteria and yeast. The IC_50_ cut-off values were set at ≤ 0.5 mg/mL for good activity and ≤0.09 mg/mL for very good activity. Among the very active extracts, methylene chloride extract of *P. strigosum* is the most representative, being very active on all Gram-positive strains [(excepted: *Staphylococcus warneri* (T26A1)], only on two Gram-negative strains [(*Stenotrophomonas maltophilia* (21170) and *Burkholderia cepacia* (13003)], and on the two *Candida albicans* strains. This extract was also quite active on *Streptococcus pyogenes* (16135). Other extracts with good activity on Gram-positive bacteria can be pointed out: methylene chloride extracts of *P. xantochyma* and *P. brasiliense*, and hydroalcoholic extract of *P. xantochyma*. Extracts with very good activity on Gram-negative bacteria are: methylene chloride extracts of *P. xantochyma* and *P. divaricatum* on *B. cepacia* (13003) and *Pseudomonas aeruginosa* (ATCC 27583), respectively. Extracts with very good activity on *Candida* strains were methylene chloride extracts of *P. pseudoarboreum*, *P. divaricatum*, and *P. heterophyllum* and hexane extract of *P. sancti-felicis*. Extracts with good activity on Gram-positive bacteria and *Candida* were principally methylene chloride of *P. brasiliense* and a hydroalcoholic extract of *P. xantochyma*.

### LCMS data mining and statistical correlation analysis

**FIGURE 1:**
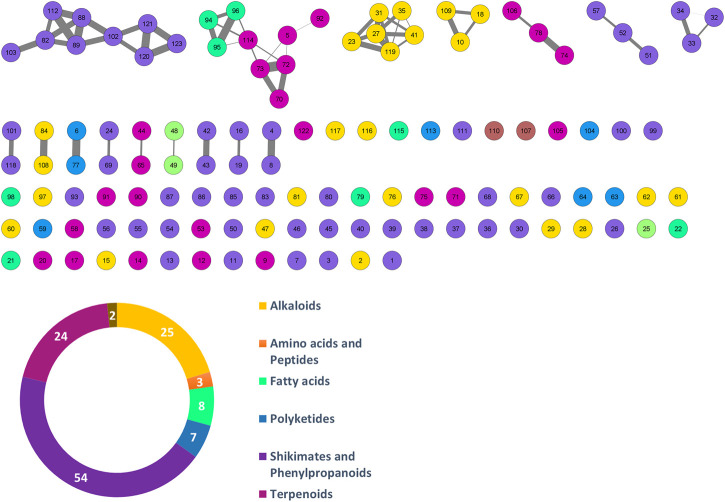
Molecular networking of putative compounds. A feature ID is allocated in each node. The colors in the network and pie chart relate to the phytochemical classes. The number of features for each class is also shown in the pie chart.

After the application MS-CleanR workflow to the LCMS data, we obtained 123 unique metabolite features (*m/z* × RT × Peak area). Among these, 56 compounds were annotated at the genus level (45.53%), 6 compounds at the family level (4.88%), and 55 compounds at the generic level (44.72%), leaving 6 compounds unannotated (4.88%) (Table 3). The MSMS fragmentation pathway of these compounds was used to establish a molecular network (Figure 1). Using NPClassifire and ClassyFire (Djoumbou Feunang et al., 2016; Kim et al., 2021), major phytochemical classes and subclasses of compounds were identified. The main classes of compounds identified were alkaloids, amino acids, fatty acids, polyketides, shikimates, phenylpropanoids, and terpenoids (metadata in Supplementary Table S3).

**TABLE 3:** Features identified as responsible for bioactivity. ^†^ = [M + H-H_2_O]^+^; ^¥^ = [M + Na]^+^; ^‡^ = based on molecular networking.

Feature ID	*m/z* [M + H]^+^	RT	Formula	Annotation	Annotation level	Phytochemical class
Group 1						
**12**	344.335	9.745	C_24_H_41_N	(1-{12,16-dimethylpentacyclo octadecan-15-yl}ethyl)dimethylamine	generic	terpenoid
**21**	362.342	9.745	C_24_H_43_NO	2,4,14-eicosatrienoic acid, 2-methylpropylamide	genus	fatty acids and conjugates
**23**	396.363	10.225	C_23_H_45_N_3_O_2_	5-(4-aminobutyl)-1,5-diazacyclohenicosane-6,14-dione	generic	alkaloids and derivatives
**27**	337.573^†^	10.391	C_26_H_47_NO	filfiline derivative	genus	alkaloids and derivatives^‡^
**31**	400.394^†^	11.035		unknown		alkaloids and derivatives^‡^
**35**	428.426	11.628		unknown		alkaloids and derivatives^‡^
**41**	456.457	12.118		unknown		alkaloids and derivatives^‡^
**45**	489.155	10.287	C_28_H_24_O_8_	ferrudiol	genus	shikimates and phenylpropanoids
**87**	271.060	9.28	C_15_H_10_O_5_	apigenin	generic	shikimates and phenylpropanoids
**95**	279.232	10.707	C_18_H_30_O_2_	gamma-linolenate	generic	fatty acids and conjugates
**119**	316.301	9.077	C_22_H37N	daphnane	generic	alkaloids and derivatives
Group 2						
**4**	151.0755	6.5	C_9_H_10_O_2_	acetanisole derivative	genus	shikimates and phenylpropanoids
**6**	221.081^¥^	8.087	C_10_H_14_O_4_	decarestrictin H	generic	polyketides
**7**	340.116^¥^	7.486	C_17_H_19_NO_5_	aduncamide	genus	shikimates and phenylpropanoids
**16**	355.118	9.49	C_20_H_18_O_6_	sesamin	genus	shikimates and phenylpropanoids
**53**	593.277^¥^	13.09	C_32_H_42_O_9_	swietenin E	generic	terpenoids
**63**	627.246	12.539	C_33_H_38_O_12_	thielavin L	generic	polyketides
**65**	197.117	6.022	C_11_H_16_O_3_	isololiolide	family	terpenoids
**77**	221.081	7.516	C_10_H_14_O_4_	modiolide A	generic	polyketides
**117**	312.159	8.705	C_19_H_21_NO_3_	piperettine	genus	alkaloids and derivatives

**FIGURE 2:**
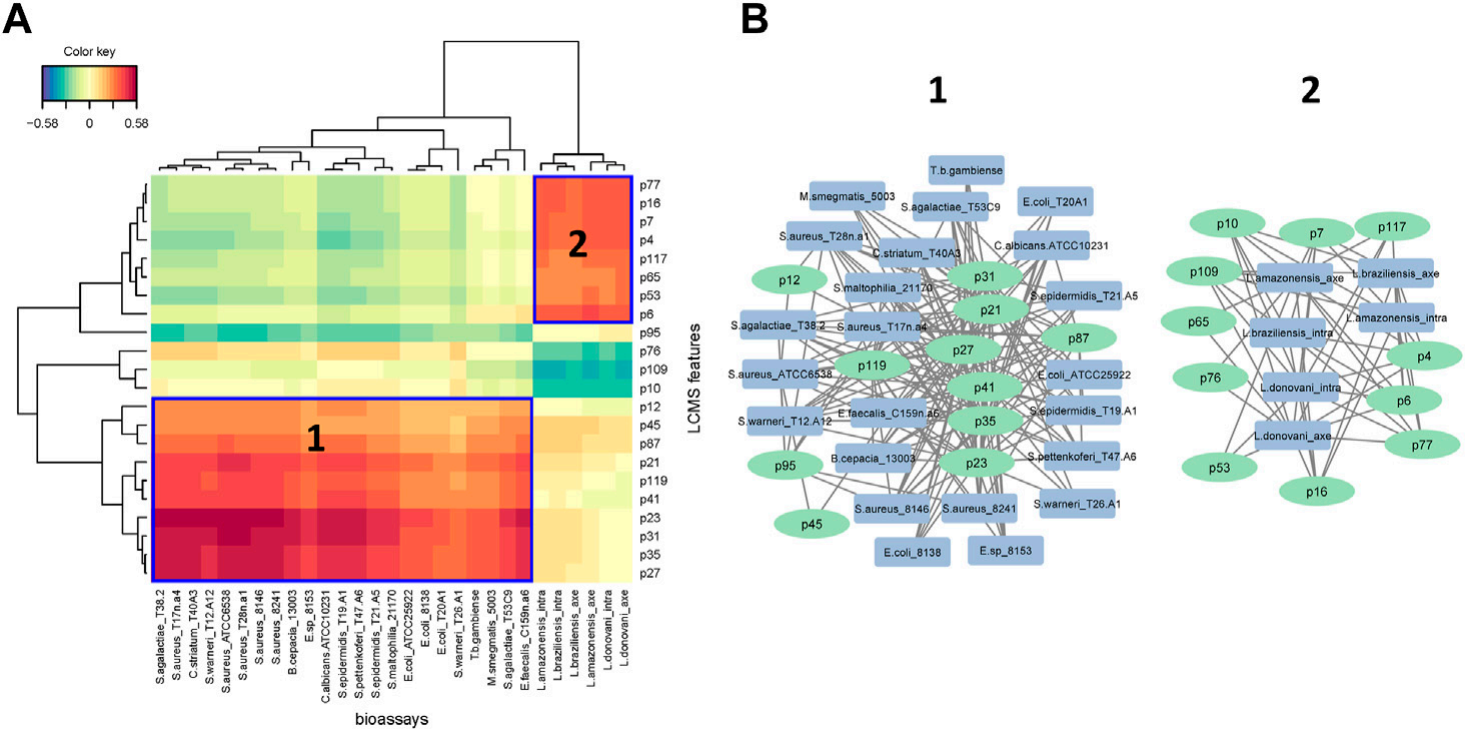
**(A)** Clustered Image Map (CIM) performed on the two-blocks of data sets. The plot depicts the correlation between X = LCMS features (p = peak) data and Y = bioassays. Variables were selected across two dimensions and clustered with a complete Euclidean distance method. Blue squares represent the two main groups of features identified by activities **(B)** The two main clusters of interaction represented by the relevance network (RN). The two analyses were tuned to a threshold of interaction of 0.3.

A heatmap of correlation analysis (Clustered Image Map, CIM) is shown in Figures 2–A. For this analysis, we compiled all *in vitro* assay results (30 bacterial strains, 2 fungal strains, 4 parasitic strains and 2 cell lines; Table 1, Supplementary Table S2) versus the LCMS features. This figure results from a setting of the threshold of correlation at 0.3 (the heatmap is shown *in extenso* in Supplemenytary Datasheet S1). Two groups of features responsible for activities can be distinguished. Group 1 is composed of 11 features (12, 21, 23, 27, 31, 35, 41, 45, 87, 95, and 119) having activity on 21 bacteria, one fungus (*C. albicans* ATCC 10231), and one parasite (*T. b. gambiense*). Activity on bacteria was mostly concentrated on Gram-positive strains [(except for *Burkholderia cepacia* (13003), *Stenotrophomonas maltophilia* (21170), and the three strains of *Escherichia coli*)]. Group 2 is composed of 9 features (4, 6, 7, 16, 53, 63, 65, 77, and 117). This group was selective on *Leishmania* (all strains, both axenic and intramacrophagic). We also performed a relevance network (RN) analysis on the same datasets to verify and complete the CIM results (Figures 2–B). RN showed two main clusters of features linked to the activity, the same as in the CIM. The LC-MSMS molecular network (Figure 1) allowed to determine that six features identified in the Group 1 belong to the alkaloids class, while others are mostly self-loops (*i.e.,* compounds not having structurally-related analogs in the extract) belonging to varied phytochemical classes. In the case of *Leishmania*-specific compounds (Group 2), they mostly belong to the polyketides or shikimates and phenylpropanoids classes, and are found only as self-loops or only related to one compound and not correlated to any activity (*i.e.*, features 16 and 4).

**FIGURE 3:**
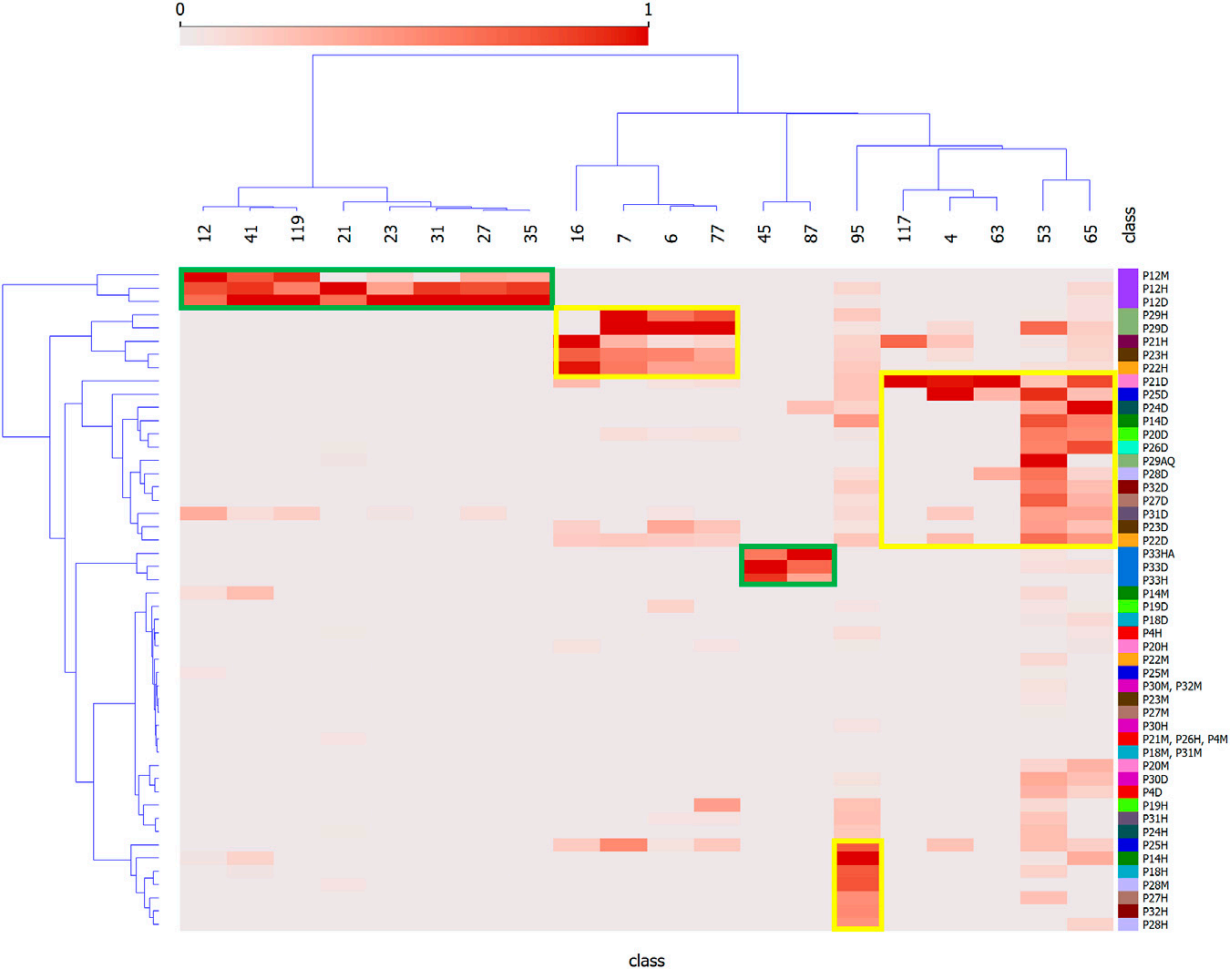
Heatmap of correlation between plant extracts and presumed bioactive peak features. Y = *Piper* extracts, and X = features of LC-MSMS analysis as shown in Figures 2A,B. Green and yellow squares represent the distribution of the bioactive features identified for the Groups 1 and 2 in the *Piper* extracts.

Figure 3 shows a heatmap depicting how the features are distributed among the extracts. Features of Group 1 are mainly distributed in all extracts of *P. strigosum* (features 12, 21, 23, 31, 41, 27 and 35) and *P. xanthostachyum* (features 45 and 87). Features of Group 2 are found in the hexane and methylene chloride extracts of *P. sancti-felicis* and hexane extracts of *P. calvescentinerve*, *P. cordatomentosa* and *P. crassinervium* (features 6, 7, 16 and 77), methylene chloride extracts of *P. pseudoarboreum*, *P. calvescentinerve, P. divaricatum*, *P. glabribaccum*, *P. bullatum*, *P. heterophyllum*, *P. reticulatum*, *P. oblongum, P. trigonum*, *P. crassinervium* and *P. cordatomentosa*, and the aqueous extract of *P. sancti-felicis* (features 4, 53, 63, 65 and 117). Feature 95 is found in the methanol and hexane extracts of *P. reticulatum* and the hexane extracts of *P. pseudoarboreum*, *P armatum*, *P. glabribaccum*, *P. oblongum* and *P. verruculosum*.

## Discussion

Plants from the *Piper* genus are currently used by the Amerindian communities in Peru in different preparations (infusion of different parts, juice directly ingested, cataplasms made of crushed leaves or stems, *etc.*) to treat different diseases (Odonne et al., 2009, 2011; Vásquez-Ocmín et al., 2021a). For the present study, we conducted ethnopharmacological surveys in Loreto and Cusco, Peruvian Amazonian departments heavily impacted by protozoal diseases. Indeed, Loreto has always been the department with the greatest number of malaria cases (principally *P. vivax*), mostly affecting children from birth to age 11 (84.5% in 2020) (Ministerio de Salud del Perú, 2021). Leishmaniasis occurrence in Peru is principally due to cutaneous (*L. amazonesis*, 17,892 cases over the last 5 years) and mucosal forms (*L. braziliensis*, *L. peruviana*, *L. lainsoni*, *L. guyanensis*, 1,642 cases in the last 5 years). To date, in 2002, 70% of the cases of both forms were concentrated in 7 departments: Madre de Dios, Cusco (Echarate and Kosñipata districts), Piura, Junín, Loreto, Cajamarca and San Martín (Ministerio de Salud del Perú, 2022). In Peru, plant-based treatments are very common and even recommended by the health personnel. It is estimated that approximately 5,000 species are used in the traditional medicine (Vásquez-Ocmín et al., 2018) among which the *Piper* species studied in this work represent 0.34%. We targeted the ethnopharmacological survey on “*Cordoncillos*” (little cords), a word used to describe pepper vines in Amazonia because the presence of nodes on the stems reminds us of a *nudo de soga* (node of cord). While this terminology would initially direct the sample collection, we took great care of properly identifying the species upon collection. In two cases, we could not fully ascertain the species and further taxonomic examination is underway.

Chemical profiling of natural extracts can be readily obtained, but for metabolomic purposes, data need to be ‘preprocessed’ (peak picking, alignment, clustering, integration and normalization) to obtain a clean dataset able to provide meaningful information upon statistical analysis. The large abundance and complexity of variables are also challenging and require to apply an appropriate statistical method to answer a specific biological question (Hervé et al., 2018). Few metabolomic studies on *Piper* species using NMR (Yamaguchi et al., 2011; Uckele et al., 2021) or LCMS have been reported so far (Vásquez-Ocmín et al., 2021a). The *Piper* genus is a good model to apply our framework for two reasons: i) *Piper* species are particularly abundant in Peru (∼260 according to www.tropicos.org), many of them used in traditional medicine; ii) many studies, including numerous phytochemical ones, were carried out on this genus (∼9,000 results found in www.webofscience.com for “*Piper*”, consulted on 1 July 2022), allowing a good metabolome coverage.

We propose in this work a computational statistical analysis to integrate two heterodimensional datasets: the features (compounds) obtained by LC-MSMS analysis of *Piper* extracts (Supplementary Table S3), and the *in vitro* biological activity of these extracts on parasites, bacteria and mammalian cells (Table 1; Supplementary Table S2), with the aim to outline most active compounds. To leverage on this multiplexed approach, we applied a *r*egularized *C*anonical *C*orrelation *A*nalysis (rCCA). This method aims to extract correlated information by maximizing their correlation via canonical variates between two datasets acquired on the same samples (vertical integration). The results of rCCA model were displayed using Clustered Image Maps (CIM) to highlight the correlation level of variables from all datasets, ordered through unsupervised clustering on both samples and compounds simultaneously (González et al., 2012). Another approach for displaying net-like correlation structures between two data sets is to use Relevance Networks (RN). This method generates a graph where nodes represent variables, and edges represent variable associations (Butte et al., 2000). Given an appropriate threshold, the RN highlights main correlation between both datasets (Figures 2–B). To our knowledge, the implementation of this type of metabolomics framework aimed at identifying bioactive compounds has not been used so far. However, a study has been recently published using rCCA to combine multiparametric analysis of adjuvanticity *in vivo* with immunological profiles *in vitro* (cytokines, chemokines, and growth factor secretion analyzed by flow cytometry) of a library of compounds derived from hot-water extracts of herbal medicines (Hioki et al., 2022).

Figure 2 strikingly shows that the antibacterial and antileishmanial activities are correlated with two distinct groups of compounds. This is by no means surprising as these organisms have significantly different physiological organization, leading to different drugs currently used against these infections. The species showing the most significant antibacterial activities are *P. strigosum* and *P. xanthostachyum*, and the species showing the most significant antileishmanial activities are *P. pseudoarboreum*, *P. calvescentinerve*, *P. glabribaccum*, *P. reticulatum* and *P. sancti-felicis*. The compounds correlated with these activities within these extracts are rather specific to these species (Figure 3). An overall weak activity of these *Piper* extracts on *P. falciparum* (Table 1) is an unexpected result, as *Piper* species are usually reputed for their antimalarial potential.

Features from Group 1 were active not only on bacteria but also on one fungus and one parasite. Although the high cross-activity observed for this group of compounds (Figure 2 and Supplementary Datasheet S1) could foretell of a lack of selectivity, these compounds did not cause cytotoxicity in mammalian cells. Six of the features of Group 1 annotated as alkaloid derivatives were grouped in a same cluster in the molecular networking, suggesting a similar biosynthesis pathway. Only three of them were annotated in our workflow, suggesting that the three unannotated ones may have an original structure. The reliability of the annotation or classification (NPClassifire and ClassyFire) part of the workflow may nevertheless be tempered, as shown by the case of feature 27, classified by the workflow as an alkaloid, but annotated as filfiline, a fatty amide previously isolated from the roots of *Piper retrofractum* (Banerji et al., 2002). The broad bioactivity spectrum of alkaloids is well known, *i.e.*, antibacterial, antiparasitic, anticancer, but also their cytotoxicity (Newman and Cragg, 2007; 2020; Daley and Cordell, 2021; Yan et al., 2021). The mechanism of action of antibacterial alkaloids is mainly described as disrupting the bacterial membrane, affecting DNA function and inhibiting protein synthesis (Ananthan et al., 2009; Pan et al., 2014; Kelley et al., 2013; Li et al., 2014; Larghi et al., 2015). Antibacterial alkaloids (MIC <10 μM/mL) are as diverse as isoquinolines, aporphines, phenanthrenes, quinolines and indoles and their activity is mostly oriented against Gram-positive bacteria (Porras et al., 2021). Quinoleines like 8-hydroxyquinoline and its derivatives and evocarpine are active on bacteria associated with respiratory system infections: *Mycobacterium tuberculosis*, *S. aureus*, and MRSA (Methicillin-resistant *Staphylococcus aureus*) (MIC ≤10 μM/mL). Aporphine alkaloid derivatives exhibit high broad-spectrum activity against Gram-positive bacteria: *S. agalactiae*, *S. aureus*, *S. epidermidis*, *E. faecalis*, and as in our study *E. coli* (Hamoud et al., 2015; Tan et al., 2015). Even if the chemical array within *Piper* spp. Is impressively diverse, nitrogen-containing compounds are limited, *i.e.*, alkaloids like piperines or amides like piplartine, phenethyls, and diaminodiamides. Amides are known to have fungicidal, cytotoxic, or antiprotozoal activities. With regard to fungicidal activity, dehydropipernonaline and nigramide R previously isolated from *P. retrofractum* displayed potent growth inhibition of *Cladosporium cladosporioides* and cytotoxicity against the L5178Y mouse lymphoma cell line (IC_50_ values of 8.9 µM and 9.3 µM, respectively) (Muharini et al., 2015). Amide piplartine isolated from a methanol extract of *P. retrofractum* showed good activity on *Leishmania donovani* with an IC_50_ value at 7.5 µM (Bodiwala et al., 2007). In Group 1, features correlated with a fungicidal activity would only target *C. albicans*. Other active compounds identified in Group 1 were one terpenoid, two fatty acids, one shikimate and phenylpropanoid derivative and one polyketide.

Compounds from the Group 2, characterized as being rather specific to *Leishmania*, are labeled as polyketides 3), shikimates and phenylpropanoids 3), terpenoids 2) and alkaloids 1). Their lack of activity on *Trypanosoma* is not surprising (Figure 2), as even though both parasites belong to the same Trypanosomatidae order, the current treatments are not similar. These two parasites cause different pathologies, in distinct geographical areas. They evolved differently and show evolutionary discrepancies in their mechanisms that may explain variations in sensitivity to treatments (Fernandes et al., 2020; Van den Broeck et al., 2020). A cross-reading of Figures 2, 3, suggests that extracts of *P. calvescentinerve* and *P. glabribaccum* and features from Group 2 have not only a selectivity for *Leishmania* but also a good selectivity index. Selectivity is a key parameter for antileishmanial drugs: antimonials, amphotericin B, paromomycin sulfate and miltefosine have variable efficacy against the *Leishmania* species but have significant adverse effects (Rao et al., 2019). Tremendous efforts have been put into the understanding of the *Leishmania* biology, leading to the identification of numerous putative targets: ergosterol and its biosynthetic pathway [*i.e.*, amphotericine B and enzymes like squalene synthase (SQS) or sterol methyltransferase (SMT)], the glycolytic pathway necessary to provide glucose as an energy source, DNA topoisomerases, enzymes of the polyamine biosynthetic pathway (*i.e.*, arginase, ornithine decarboxylase, s-adenosylmethionine decarboxylase, and spermidine synthase), redox metabolism pathway (Nagle et al., 2014; Raj et al., 2020).

The compounds labeled as shikimates or phenylpropanoids (acetanisole, aduncamide, and sesamin) or alkaloid (piperettine) were annotated at the genus level because they had previously been isolated from *Piper tuberculatum*, *Piper nigrum*, *P. longum*, *Piper aduncum*, *Piper puberulum*, *P. austrosinense*, *Piper brachystachyum*, *P. mullesua*, *P. retrofractum*, *P. sarmentosum* and *P. sylvaticum* (Spring and Stark, 1950; Orjala et al., 1993; De Araujo-Junior et al., 1997; Puri et al., 1998; Umezawa, 2003; Tuntiwachwuttikul et al., 2006). Among these compounds, only the lignan sesamin was reported to show activity on *L. amazonensis* with an IC_50_ of 44.6 μM/mL and was not cytotoxic for mouse macrophage cells (CC_50_ > 100 μg/mL, SI > 6) (Pulivarthi et al., 2015). A study based on computational methods suggests that sesamin could be a promising inhibitor of the *L. donovani* CRK12 receptor (binding affinity of −8.5 kcal/mol) (Broni et al., 2021). Nevertheless, sesamin, isolated from a hexane extract of *P. retrofractum* (IC_50_ of the extract = 5 μg/mL), was inactive on *L. donovani* (Bodiwala et al., 2007) (Bodiwala et al., 2007). This compound was also inactive on *Plasmodium falciparum* K1 multidrug resistant strain*, M. tuberculosis* H37Ra, and *Candida albicans* (EC_50_ > 20 μg/mL, MIC >200 μg/mL and IC_50_ > 50 μg/mL, respectively). Cubein, another lignan isolated from *Piper cubeba*, was shown to be active on *L. donovani* (IC_50_ = 28.0 µM). Interestingly, sesamin is connected in the MN with an unknown compound (RT = 6.577 [M + H]^+^ = 360.145). Piperettine has been previously isolated from *P. nigrum* and *P. aurantiacum* and was shown to be active on epimastigotes and amastigotes of *Trypanosoma cruzi* (IC_50_ = 10.67 and 7.40 µM, respectively) (Ribeiro et al., 2004). In our work, the compound annotated as piperettine was only detected in *P. calvescentinerve* extracts (Figure 3) but was not labeled as being correlated with any activity on *T b. gambiense* (Supplementary Datasheet S1). The use of this *Piper* species as a medicinal plant with various indications can partly be validated by the fact that it is an inhibitor of 5-lipoxygenase (76.02 µM) (Muthuraman et al., 2019), a key enzyme involved in the biosynthesis of pro-inflammatory leukotrienes, provided its concentration is sufficient in the traditional preparations. Aduncamide was shown to present a moderate antineuroinflammatory activity (IC_50_ at 26 ± 8.3 µM) by the Griess method on LPS-stimulated BV-2 cells (Zheng et al., 2021), to be cytotoxic for KB nasopharyngeal carcinoma cells (ED_50_ = 5.7 μg/mL) and to inhibit growth Gram-positive *Burkholderia subtilis* and *Mycobacterium luteus*, while being less active towards Gram-negative *E. coli* (Orjala et al., 1993). In our work, the compound annotated as aduncamide was identified in extracts of *P. glabribaccum*, *P. calvescentinerve* and *P. cordatomentosa*, whose extracts are among the most active on *Leishmania*. Two compounds were labeled as 10-membered lactone macrolides and annotated as decarestrictin H and modiolide A, previously isolated from the fungi *Penicillium simplicissimum* and *Stagonospora cirsii*, respectively (Grabley et al., 1992; Evidente et al., 2008). These two last annotations, although resulting from similar fragmentation patterns, may be subject to caution. Indeed, these compounds were annotated at the generic level and no macrolide has been identified in *Piper* spp. so far (only a macrolactam, laevicarpin, previously isolated from *P. laevicarpus*) (da Silva A. Maciel et al., 2016). Furthermore, macrolides are well-known antibacterial compounds and thus should logically rather be clustered in Group 1, if ever present in the extracts. A compound annotated as apigenin was detected only in *P. xanthostachyum* and was identified as being correlated to the antibacterial activity (Figure 2A). This may appear surprising given that apigenin is a common and ubiquitous flavonoid with no antibacterial activity. The number of possible flavonoid isomers being quite large, this annotation may also be subject to caution, notwithstanding the fact that the actual compound (p87) being present in the extract remains of interest from the antibacterial perspective. Other compounds were annotated as flavonoids in the extracts (most of the compounds in Group 3, see Supplementary Datasheet S1), characterized by a moderate activity of the extracts containing them, a moderate correlation with all the activities without any clear selectivity. These compounds were annotated at the genus level, as they were previously isolated from *Piper* species (Matsui and Munakata, 1976; Lago et al., 2004; González et al., 2022). Antiprotozoal or antimicrobial activity of flavonoids is well documented (Graf et al., 2005; Ortiz et al., 2017; 2020). Two compounds were annotated as pinocembrine and pinostrobin, both belonging to the flavanones subclass. Pinocembrine displays antifungal (*C. cladosporioides* and *C. sphaerospermum*) or antibacterial (*Enterococcus faecalis*, *M. tuberculosis*) potential (Lago et al., 2004; Jeong et al., 2009; Gröblacher et al., 2012), while showing either no cytotoxicity on healthy and cancerous cell lines (RAW 264.7, epidermoid carcinoma of the oral cavity (KB), human small cell lung cancer (NCI-H187), metastatic murine colon 26-L5 carcinoma, PANC-1 human pancreatic cancer, metastatic human HT-1080 fibrosarcoma), or high cytotoxicity (Awale et al., 2009; Yenjai and Wanich, 2010; Lee et al., 2013). Pinostrobin showed weak antimalaria activity on *P. falciparum* (Kaur et al., 2009) but significant potential as a cancer chemopreventive agent (Gu et al., 2002).

Some of these *Piper* compounds are therefore of interest in an isolation and factual testing perspective. Compounds annotated as aduncamide, sesamin, and apigenin, along with the unknown compounds correlated to the activity, could be subjected to mass-targeted isolation (Vásquez-Ocmín et al., 2022), in order to confirm their annotation or identify their structure, as well as confirm their biological potential.

## Conclusion

Hyphenated analytical techniques are very useful to provide highly informative chemical profiling of complex metabolomes. Nevertheless, deciphering such profiles to determine the compounds responsible for the biological activity remains a challenge. By correlating these chemical profiles with biological assay results, we propose a workflow of integrated metabolomics statistical tools to provide a broad picture of the metabolome as well as a map of compounds putatively associated to the bioactivity. The structure of these compounds can either be annotated from previous works or remain unknown at this stage, but in any case, they require a formal isolation step to confirm their structure and activity. Nevertheless, an initial data mining on analytical-scale data proves very effective for prioritizing the compounds to target. The relevance of our approach has been validated on a set of *Piper* species tested for their anti-infectious diseases at the extract level. Such an approach can be extended to any type of natural extract, particularly when prior phytochemical data is available in the literature.

## Data Availability

The original contributions presented in the study are included in the article/Supplementary Material, further inquiries can be directed to the corresponding authors.

## References

[B1] Alarcon-Barrera J. C. Kostidis S. Ondo-Mendez A. Giera M. (2022). Recent advances in metabolomics analysis for early drug development. Drug Discov. Today 27, 1763–1773. 10.1016/j.drudis.2022.02.018 35218927

[B2] Awale S. Miyamoto T. Linn T. Z. Li F. Win N. N. Tezuka Y. (2009). Cytotoxic constituents of *Soymida febrifuga* from Myanmar. J. Nat. Prod. 72, 1631–1636. 10.1021/np9003323 19689125

[B3] Balaraman K. Vieira N. C. Moussa F. Vacus J. Cojean S. Pomel S. (2015). *In vitro* and *in vivo* antileishmanial properties of a 2-n-propylquinoline hydroxypropyl β-cyclodextrin formulation and pharmacokinetics via intravenous route. Biomed. Pharmacother. 76, 127–133. 10.1016/j.biopha.2015.10.028 26653559

[B4] Banerji A. Sarkar M. Datta R. Sengupta P. Abraham K. (2002). Amides from *Piper brachystachyum* and *Piper retrofractum* . Phytochemistry 59, 897–901. 10.1016/S0031-9422(01)00364-8 11937173

[B5] Bocquet L. Sahpaz S. Bonneau N. Beaufay C. Mahieux S. Samaillie J. (2019). Phenolic compounds from *Humulus lupulus* as natural antimicrobial products: New weapons in the fight against methicillin resistant *Staphylococcus aureus, Leishmania mexicana* and *Trypanosoma brucei* Strains. Molecules 24, E1024. 10.3390/molecules24061024 PMC6472001 30875854

[B6] Bodiwala H. S. Singh G. Singh R. Dey C. S. Sharma S. S. Bhutani K. K. (2007). Antileishmanial amides and lignans from *Piper cubeba* and *Piper retrofractum* . J. Nat. Med. 61, 418–421. 10.1007/s11418-007-0159-2

[B7] Broni E. Kwofie S. K. Asiedu S. O. Miller W. A. Wilson M. D. (2021). A molecular modeling approach to identify potential antileishmanial compounds against the cell division cycle (cdc)-2-related kinase 12 (CRK12) receptor of *Leishmania donovani* . Biomolecules 11, 458. 10.3390/biom11030458 33803906 PMC8003136

[B8] Butte A. J. Tamayo P. Slonim D. Golub T. R. Kohane I. S. (2000). Discovering functional relationships between RNA expression and chemotherapeutic susceptibility using relevance networks. Proc. Natl. Acad. Sci. 97, 12182–12186. 10.1073/pnas.220392197 11027309 PMC17315

[B9] CLSI (2006). Methods for dilution antimicrobial susceptibility test for bacteria that grow aerobically, aproved standard. Available at: https://infostore.saiglobal.com/en-us/standards/CLSI-M7-A7-7ED-2006-357843_SAIG_CLSI_CLSI_815015/ (Accessed September 2, 2022).

[B10] da Silva A. Maciel D. Freitas V. P. Conserva G. A. A. Alexandre T. R. Purisco S. U. (2016). Bioactivity-guided isolation of laevicarpin, an antitrypanosomal and anticryptococcal lactam from *Piper laevicarpu* (Piperaceae). Fitoterapia 111, 24–28. 10.1016/j.fitote.2016.04.005 27083380

[B11] Daley S. Cordell G. A. (2021). Alkaloids in contemporary drug discovery to meet global disease needs. Molecules 26, 3800. 10.3390/molecules26133800 34206470 PMC8270272

[B12] De Araujo-Junior J. X. Da-Cunha E. V. L. Chaves M. C. D. O. Gray A. I. (1997). Piperdardine, a piperidine alkaloid from *Piper tuberculatum* . Phytochemistry 44, 559–561. 10.1016/S0031-9422(96)00503-1

[B13] de Salud del Perú M. (2022). Boletín Epidemiológico del Perú-Situación epidemiológica de la Leishmaniasis en el Perú SE 12-2022. Available at: https://www.dge.gob.pe/epipublic/uploads/boletin/boletin_202212_22_181950_2.pdf (Accessed October 2, 2021).

[B14] Djoumbou Feunang Y. Eisner R. Knox C. Chepelev L. Hastings J. Owen G. (2016). ClassyFire: Automated chemical classification with a comprehensive, computable taxonomy. J. Cheminformatics 8, 61. 10.1186/s13321-016-0174-y PMC5096306 27867422

[B15] Durant-Archibold A. A. Santana A. I. Gupta M. P. (2018). Ethnomedical uses and pharmacological activities of most prevalent species of genus *piper* in Panama: A review. J. Ethnopharmacol. 217, 63–82. 10.1016/j.jep.2018.02.008 29428241

[B16] Evidente A. Cimmino A. Berestetskiy A. Andolfi A. Motta A. (2008). Stagonolides G−I and Modiolide A, nonenolides produced by *Stagonospora cirsii*, a potential mycoherbicide for *Cirsium arvense* . J. Nat. Prod. 71, 1897–1901. 10.1021/np800415w 18959441

[B17] Fernandes P. M. Kinkead J. McNae I. W. Vásquez‐Valdivieso M. Wear M. A. Michels P. A. M. (2020). Kinetic and structural studies of *Trypanosoma* and *Leishmania* phosphofructokinases show evolutionary divergence and identify AMP as a switch regulating glycolysis versus gluconeogenesis. FEBS J. 287, 2847–2861. 10.1111/febs.15177 31838765 PMC7383607

[B18] Fraisier-Vannier O. Chervin J. Cabanac G. Puech V. Fournier S. Durand V. (2020). MS-CleanR: A feature-filtering workflow for untargeted LC–MS based metabolomics. Anal. Chem. 92, 9971–9981. 10.1021/acs.analchem.0c01594 32589017

[B19] González A. S. Tellini V. H. S. Gutiérrez D. M. B. (2022). Study of the dermal anti-inflammatory, antioxidant, and analgesic activity of pinostrobin. Heliyon 8, e10413. 10.1016/j.heliyon.2022.e10413 36097473 PMC9463643

[B20] González I. Cao K.-A. L. Davis M. J. Déjean S. (2012). Visualising associations between paired ‘omics’ data sets. BioData Min. 5, 19. 10.1186/1756-0381-5-19 23148523 PMC3630015

[B21] González I. Déjean S. Martin P. G. P. Baccini A. (2008). Cca: An R package to extend canonical correlation analysis. J. Stat. Softw. 23, 1–14. 10.18637/jss.v023.i12

[B22] Grabley S. Hammann P. Hütter K. Kirsch R. Kluge H. Thiericke R. (1992). Secondary metabolites by chemical screening 20 decarestrictines, a new family of inhibitors of cholesterol biosynthesis from *Penicillium* III. Decarestrictines E M. J. Antibiot. 45, 1176–1181. 10.7164/antibiotics.45.1176 1517163

[B23] Graf B. A. Milbury P. E. Blumberg J. B. (2005). Flavonols, flavones, flavanones, and human health: Epidemiological evidence. J. Med. Food 8, 281–290. 10.1089/jmf.2005.8.281 16176136

[B24] Gröblacher B. Kunert O. Bucar F. (2012). Compounds of *Alpinia katsumadai* as potential efflux inhibitors in *Mycobacterium smegmatis* . Bioorg. Med. Chem. 20, 2701–2706. 10.1016/j.bmc.2012.02.039 22459211

[B25] Gu J.-Q. Park E. J. Vigo J. S. Graham J. G. Fong H. H. S. Pezzuto J. M. (2002). Activity-guided isolation of constituents of *Renealmia nicolaioides* with the potential to induce the hhase II enzyme quinone reductase. J. Nat. Prod. 65, 1616–1620. 10.1021/np020249p 12444686

[B26] Hamoud R. Reichling J. Wink M. (2015). Synergistic antibacterial activity of the combination of the alkaloid sanguinarine with EDTA and the antibiotic streptomycin against multidrug resistant bacteria. J. Pharm. Pharmacol. 67, 264–273. 10.1111/jphp.12326 25495516

[B27] Hervé M. R. Nicolè F. Lê Cao K.-A. (2018). Multivariate analysis of multiple datasets: A practical guide for chemical ecology. J. Chem. Ecol. 44, 215–234. 10.1007/s10886-018-0932-6 29479643

[B28] Hioki K. Hayashi T. Natsume-Kitatani Y. Kobiyama K. Temizoz B. Negishi H. (2022). Machine learning-assisted screening of herbal medicine extracts as vaccine adjuvants. Front. Immunol. 13. Available at: https://www.frontiersin.org/articles/10.3389/fimmu.2022.847616 (Accessed September 7, 2022). 10.3389/fimmu.2022.847616 PMC9160479 35663999

[B29] Jeong K.-W. Lee J.-Y. Kang D.-I. Lee J.-U. Shin S. Y. Kim Y. (2009). Screening of flavonoids as candidate antibiotics against *Enterococcus faecalis* . J. Nat. Prod. 72, 719–724. 10.1021/np800698d 19236029

[B30] Kaur K. Jain M. Kaur T. Jain R. (2009). Antimalarials from nature. Bioorg. Med. Chem. 17, 3229–3256. 10.1016/j.bmc.2009.02.050 19299148

[B31] Kelley C. Lu S. Parhi A. Kaul M. Pilch D. S. LaVoie E. J. (2013). Antimicrobial activity of various 4- and 5-substituted 1-phenylnaphthalenes. Eur. J. Med. Chem. 60, 395–409. 10.1016/j.ejmech.2012.12.027 23314053 PMC3949621

[B32] Kim H. W. Wang M. Leber C. A. Nothias L.-F. Reher R. Kang K. B. (2021). NPClassifier: A deep neural network-based structural classification tool for natural products. J. Nat. Prod. 84, 2795–2807. 10.1021/acs.jnatprod.1c00399 34662515 PMC8631337

[B33] Lago J. H. G. Ramos C. S. Casanova D. C. C. Morandim A. de A. Bergamo D. C. B. Cavalheiro A. J. (2004). Benzoic acid derivatives from *Piper* species and their fungitoxic activity against *Cladosporium cladosporioides* and *C. sphaerospermum* . J. Nat. Prod. 67, 1783–1788. 10.1021/np030530j 15568762

[B34] Lambros C. Vanderberg J. P. (1979). Synchronization of Plasmodium falciparum erythrocytic stages in culture. J. Parasitol. 65, 418–420. 10.2307/3280287 383936

[B35] Larghi E. L. Bracca A. B. J. Aguilar A. A. A. Heredia D. A. Pergomet J. L. Simonetti S. O. (2015). Neocryptolepine: A promising indoloisoquinoline alkaloid with interesting biological activity. Evaluation of the drug and its most relevant analogs. Curr. Top. Med. Chem. 15, 1683–1707. 10.2174/1568026615666150427113937 25915612

[B36] Le Cao K.-A. Rohart F. Gonzalez I. Dejean S. Gautier B. Bartolo F. (2016). mixOmics: Omics data integration project. R package version 6.1.1. Available at: https://CRAN.R-project.org/package=mixOmics.

[B37] Lee C. Lee J. W. Jin Q. Jang D. S. Lee S.-J. Lee D. (2013). Inhibitory constituents of the heartwood of *Dalbergia odorifera* on nitric oxide production in RAW 264.7 macrophages. Bioorg. Med. Chem. Lett. 23, 4263–4266. 10.1016/j.bmcl.2013.04.032 23743282

[B38] Li N. Tan S. Cui J. Guo N. Wang W. Zu Y. (2014). PA-1, a novel synthesized pyrrolizidine alkaloid, inhibits the growth of *Escherichia coli* and *Staphylococcus aureus* by damaging the cell membrane. J. Antibiot. 67, 689–696. 10.1038/ja.2014.49 24894184

[B39] Matsui K. Munakata K. (1976). Four new neolignans from *Piper futokadzura* . Tetrahedron Lett. 17, 4371–4374. 10.1016/0040-4039(76)80118-9

[B40] Mgbeahuruike E. E. Yrjönen T. Vuorela H. Holm Y. (2017). Bioactive compounds from medicinal plants: Focus on Piper species. South Afr. J. Bot. 112, 54–69. 10.1016/j.sajb.2017.05.007

[B41] Ministerio de Salud del Perú (2021). Boletín Epidemiológico del Perú SE 02-2021. Available at: https://cdn.www.gob.pe/uploads/document/file/1865086/Bolet%C3%ADn%20epidemiol%C3%B3gico%20del%20Per%C3%BA%202021.pdf?v=1629922432 (Accessed October 2, 2021).

[B42] Muharini R. Liu Z. Lin W. Proksch P. (2015). New amides from the fruits of *Piper retrofractum* . Tetrahedron Lett. 56, 2521–2525. 10.1016/j.tetlet.2015.03.116

[B43] Muthuraman S. Sinha S. Vasavi C. S. Waidha K. M. Basu B. Munussami P. (2019). Design, synthesis and identification of novel coumaperine derivatives for inhibition of human 5-LOX: Antioxidant, pseudoperoxidase and docking studies. Bioorg. Med. Chem. 27, 604–619. 10.1016/j.bmc.2018.12.043 30638966

[B44] Nagle A. S. Khare S. Kumar A. B. Supek F. Buchynskyy A. Mathison C. J. N. (2014). Recent developments in drug discovery for leishmaniasis and human african trypanosomiasis. Chem. Rev. 114, 11305–11347. 10.1021/cr500365f 25365529 PMC4633805

[B45] Newman D. J. Cragg G. M. (2007). Natural products as sources of new drugs over the last 25 years. J. Nat. Prod. 70, 461–477. 10.1021/np068054v 17309302

[B46] Newman D. J. Cragg G. M. (2020). Natural products as sources of new drugs over the nearly four decades from 01/1981 to 09/2019. J. Nat. Prod. 83, 770–803. 10.1021/acs.jnatprod.9b01285 32162523

[B47] Olivon F. Elie N. Grelier G. Roussi F. Litaudon M. Touboul D. (2018). MetGem software for the generation of molecular networks based on the t-SNE algorithm. Anal. Chem. 90, 13900–13908. 10.1021/acs.analchem.8b03099 30335965

[B48] Orjala J. Wright A. D. Rali T. Sticher O. (1993). Aduncamide, a cytotoxic and antibacterial b-phenylethylamine-derived amide from *Piper aduncum* . Nat. Product. Lett. 2, 231–236. 10.1080/10575639308043814

[B49] Ortiz S. Dali-Yahia K. Vásquez-Ocmín P. Grougnet R. Grellier P. Michel S. (2017). Heme-binding activity of methoxyflavones from *Pentzia monodiana* maire (asteraceae). Fitoterapia 118, 1–5. 10.1016/j.fitote.2017.01.012 28167052

[B50] Ortiz S. Vásquez-Ocmín P. G. Cojean S. Bouzidi C. Michel S. Figadère B. (2020). Correlation study on methoxylation pattern of flavonoids and their heme-targeted antiplasmodial activity. Bioorg. Chem. 104, 104243. 10.1016/j.bioorg.2020.104243 32920360

[B51] Pomel S. Dubar F. Forge D. Loiseau P. M. Biot C. (2015). New heterocyclic compounds: Synthesis and antitrypanosomal properties. Bioorg. Med. Chem. 23, 5168–5174. 10.1016/j.bmc.2015.03.029 25835356

[B52] Porras G. Chassagne F. Lyles J. T. Marquez L. Dettweiler M. Salam A. M. (2021). Ethnobotany and the role of plant natural products in antibiotic drug discovery. Chem. Rev. 121, 3495–3560. 10.1021/acs.chemrev.0c00922 33164487 PMC8183567

[B53] Pulivarthi D. Steinberg K. M. Monzote L. Piñón A. Setzer W. N. (2015). Antileishmanial activity of compounds isolated from *Sassafras albidum* . Nat. Prod. Commun. 10, 1934578X1501000–1230. 10.1177/1934578x1501000723 26411017

[B54] Puri B. Hall A. Harborne J. B. Baxter H. Moss G. P. (1998). “Lignans,” in Phytochemical dictionary (London: CRC Press).

[B55] Raj S. Sasidharan S. Balaji S. N. Saudagar P. (2020). An overview of biochemically characterized drug targets in metabolic pathways of *Leishmania* parasite. Parasitol. Res. 119, 2025–2037. 10.1007/s00436-020-06736-x 32504119

[B56] Rao S. P. S. Barrett M. P. Dranoff G. Faraday C. J. Gimpelewicz C. R. Hailu A. (2019). Drug discovery for Kinetoplastid diseases: Future directions. ACS Infect. Dis. 5, 152–157. 10.1021/acsinfecdis.8b00298 30543391

[B57] Ribeiro T. S. Freire-de-Lima L. Previato J. O. Mendonça-Previato L. Heise N. Freire de Lima M. E. (2004). Toxic effects of natural piperine and its derivatives on epimastigotes and amastigotes of *Trypanosoma cruzi* . Bioorg. Med. Chem. Lett. 14, 3555–3558. 10.1016/j.bmcl.2004.04.019 15177472

[B58] Ruiz-Vásquez L. Ruiz Mesia L. Caballero Ceferino H. D. Ruiz Mesia W. Andrés M. F. Díaz C. E. (2022). Antifungal and herbicidal potential of *Piper* essential oils from the Peruvian Amazonia. Plants 11, 1793. 10.3390/plants11141793 35890427 PMC9324010

[B59] Salehi B. Zakaria Z. A. Gyawali R. Ibrahim S. A. Rajkovic J. Shinwari Z. K. (2019). Piper species: A comprehensive review on their phytochemistry, biological activities and applications. Molecules 24, 1364. 10.3390/molecules24071364 30959974 PMC6479398

[B60] SFE (2022). Ethnopharmacologie, plantes médicinales, médecine traditionnelle. Société Française d’Ethnopharmacologie. Available at: http://www.ethnopharmacologia.org/definition/ (Accessed July 26, 2022).

[B61] Shannon P. Markiel A. Ozier O. Baliga N. S. Wang J. T. Ramage D. (2003). Cytoscape: A software environment for integrated models of biomolecular interaction networks. Genome Res. 13, 2498–2504. 10.1101/gr.1239303 14597658 PMC403769

[B62] Spring F. S. Stark J. (1950). Piperettine from *Piper nigrum*; its isolation, identification, and synthesis. J. Chem. Soc., 1177–1180. 10.1039/JR9500001177

[B63] Tan K. K. Khoo T. J. Rajagopal M. Wiart C. (2015). Antibacterial alkaloids from *Artabotrys crassifolius* Hook.f. & thomson. Nat. Prod. Res. 29, 2346–2349. 10.1080/14786419.2015.1013954 25738993

[B64] Trager W. Jensen J. B. (1976). Human malaria parasites in continuous culture. Science 193, 673–675. 10.1126/science.781840 781840

[B65] Trujillo W. Trujillo E. T. Ortiz-Morea F. A. Toro D. A. Jaramillo M. A. (2022). New *Piper* species from the eastern slopes of the Andes in northern South America. PhytoKeys 206, 25–48. 10.3897/phytokeys.206.75971 36761273 PMC9848990

[B66] Tsugawa H. Cajka T. Kind T. Ma Y. Higgins B. Ikeda K. (2015). MS-DIAL: Data-independent MS/MS deconvolution for comprehensive metabolome analysis. Nat. Methods 12, 523–526. 10.1038/nmeth.3393 25938372 PMC4449330

[B67] Tsugawa H. Kind T. Nakabayashi R. Yukihira D. Tanaka W. Cajka T. (2016). Hydrogen rearrangement rules: Computational MS/MS fragmentation and structure elucidation using MS-FINDER software. Anal. Chem. 88, 7946–7958. 10.1021/acs.analchem.6b00770 27419259 PMC7063832

[B68] Tuntiwachwuttikul P. Phansa P. Pootaeng-on Y. Taylor W. C. (2006). Chemical constituents of the roots of *piper sarmentosum* . Chem. Pharm. Bull. 54, 149–151. 10.1248/cpb.54.149 16462055

[B69] Uckele K. A. Jahner J. P. Tepe E. J. Richards L. A. Dyer L. A. Ochsenrider K. M. (2021). Phytochemistry reflects different evolutionary history in traditional classes versus specialized structural motifs. Sci. Rep. 11, 17247. 10.1038/s41598-021-96431-3 34446754 PMC8390663

[B70] Umezawa T. (2003). Diversity in lignan biosynthesis. Phytochem. Rev. 2, 371–390. 10.1023/B:PHYT.0000045487.02836.32

[B71] Van den Broeck F. Savill N. J. Imamura H. Sanders M. Maes I. Cooper S. (2020). Ecological divergence and hybridization of Neotropical *Leishmania* parasites. Proc. Natl. Acad. Sci. U. S. A. 117, 25159–25168. 10.1073/pnas.1920136117 32958676 PMC7547230

[B72] Vásquez-Ocmín P. Cojean S. Rengifo E. Suyyagh-Albouz S. Amasifuen Guerra C. A. Pomel S. (2018). Antiprotozoal activity of medicinal plants used by Iquitos-Nauta road communities in Loreto (Peru). J. Ethnopharmacol. 210, 372–385. 10.1016/j.jep.2017.08.039 28887215

[B73] Vásquez-Ocmín P. G. Gadea A. Cojean S. Marti G. Pomel S. Van Baelen A.-C. (2021a). Metabolomic approach of the antiprotozoal activity of medicinal *Piper* species used in Peruvian Amazon. J. Ethnopharmacol. 264, 113262. 10.1016/j.jep.2020.113262 32818574

[B74] Vásquez-Ocmín P. G. Gallard J.-F. Van Baelen A.-C. Leblanc K. Cojean S. Mouray E. (2022). Biodereplication of antiplasmodial extracts: Application of the amazonian medicinal plant piper coruscans kunth. Molecules 27, 7638. 10.3390/molecules27217638 36364460 PMC9656727

[B75] Vásquez-Ocmín P. G. Marti G. Bonhomme M. Mathis F. Fournier S. Bertani S. (2021b). Cannabinoids vs. whole metabolome: Relevance of cannabinomics in analyzing *Cannabis* varieties. Anal. Chim. Acta 1184, 339020. 10.1016/j.aca.2021.339020 34625242

[B76] Vásquez-Ocmín P. G. Marti G. Gadea A. Cabanac G. Vásquez-Briones J. A. Casavilca-Zambrano S. (2023). Metabotyping of Andean pseudocereals and characterization of emerging mycotoxins. Food Chem. 407, 135134. 10.1016/j.foodchem.2022.135134 36527946

[B77] Vásquez-Ocmín P. Haddad M. Gadea A. Jullian V. Castillo D. Paloque L. (2017). A new phthalide derivative from *Peperomia nivalis* . Nat. Prod. Res. 31, 138–142. 10.1080/14786419.2016.1219857 27561759

[B78] WHO (2022a). Neglected tropical diseases. Available at: https://www.who.int/health-topics/neglected-tropical-diseases (Accessed July 25, 2022).

[B79] WHO (2022b). The sustainable development goals. Available at: https://www.un.org/sustainabledevelopment/ (Accessed October 3, 2022).

[B80] WHO (2021). World malaria report 2021. Available at: https://www.who.int/teams/global-malaria-programme/reports/world-malaria-report-2021 (Accessed July 26, 2022).

[B81] Yamaguchi L. F. Freitas G. C. Yoshida N. C. Silva R. A. Gaia A. M. Silva A. M. (2011). Chemometric analysis of ESIMS and NMR data from *Piper* species. J. Braz. Chem. Soc. 22, 2371–2382. 10.1590/S0103-50532011001200019

[B82] Yan Y. Li X. Zhang C. Lv L. Gao B. Li M. (2021). Research progress on antibacterial activities and mechanisms of natural alkaloids: A review. Antibiotics 10, 318. 10.3390/antibiotics10030318 33808601 PMC8003525

[B83] Yenjai C. Wanich S. (2010). Cytotoxicity against KB and NCI-H187 cell lines of modified flavonoids from *Kaempferia parviflora* . Bioorg. Med. Chem. Lett. 20, 2821–2823. 10.1016/j.bmcl.2010.03.054 20362442

[B84] Zheng Y. Su B. Wang Y. Wang H. Liao H. Liang D. (2021). New tyramine- and aporphine-type alkamides with NO release inhibitory activities from *Piper puberulum* . J. Nat. Prod. 84, 1316–1325. 10.1021/acs.jnatprod.1c00055 33822610

