# Supplementary material for: Deciphering anti-infectious compounds from Peruvian medicinal Cordoncillos extract library through multiplexed assays and chemical profiling
Source: Front Pharmacol. 2023 Jun 5;14:1100542. doi: 10.3389/fphar.2023.1100542 (PMC10278888; doi:10.3389/fphar.2023.1100542)

Supplementary Information

**Supporting information 1.** Set samples and description of *Piper* species

| **Date of collection** | **Species** | **Part extracted** | **Voucher number** | **UTM coordinates** | **Place of collection, department** |
| --- | --- | --- | --- | --- | --- |
|  |  |  |  |  |  |
| 14/12/2020 | *Piper casapiense* (Miq.) C. DC. | Leaves and stems | 041044 | 755672  8111253 | Estación Isula-Rio Napo, Lago Sunimiraño, Loreto |
| 14/12/2020 | *Piper strigosum* Trel. | Leaves and stems | 029675 | 513164,15  8111231 | Estación Isula-Rio Napo, Loreto |
| 01/08/2020 | *Piper pseudoarboreum* Yunck | Leaves and stems | 019724 | 526944  8141995 | Carretera Iquitos Nauta km 29, distrito de San Juan Bautista, Loreto |
| 27/11/2020 | *Piper armatum* Trel. & Yunck | Leaves and stems | 024563 | 527171  8118136 | Comunidad del gallito, Rio Amazonas, Loreto |
| 11/12/2010 | *Piper brasiliense* C. DC | Leaves and stems | 033310 | 0711335 9623600 | Estación Isula-Rio Napo, Lago Sunimiraño, Loreto |
| 6/12/2020 | *“Piper bullatum* Vahl” | Leaves and stems | 23129 | 295957  8557659 | Ámbito del valle de Kosñipata, distrito de Kosñipata, Provincia San Pedro, Cusco |
| 6/12/2020 | *Piper calvescentinerve* Trel | Leaves and stems | 23127 | 233321  8559681 | Ámbito del valle de Kosñipata, distrito de Kosñipata, Provincia San Pedro, Chontachaca, Cusco |
| 6/12/2020 | *“Piper cordatomentosa"* | Leaves and stems | 23125 | 233321  8559681 | Ámbito del valle de Kosñipata, distrito de Kosñipata, Provincia San Pedro, Chontachaca, Cusco |
| 6/12/2020 | *Piper crassinervium* Kunth. | Leaves and stems | 23128 | 295957  8557659 | Ámbito del valle de Kosñipata, distrito de Kosñipata, Provincia San Pedro, Chontachaca, Cusco |
| 22/09/2010 | *Piper divaricatum* G. Mey | Leaves | 10538 | 0288151  9312640 | Comunidad nuevo Cutervo, distrito Jepelacio, San Martin |
| 6/12/2020 | *Piper glabribaccum* Trel | Leaves and stems | 23121 | 215490  8544039 | Ámbito del valle de Kosñipata, San Pedro, Cusco |
| 20/12/2009 | *Piper heterophyllum* Ruiz & Pav. | Leaves | 028164 | 513560  8111901 | Carretera Mazan-Indiana, Loreto. |
| 6/12/2020 | *Piper oblongum* Kunth | Leaves and stems | 23123 | 231733  8559990 | Ámbito del valle de Kosñipata, distrito de Kosñipata, Provincia San Pedro, Buenos aires, Cuzco. |
| 11/01/2010 | *Piper reticulatum* L | Leaves | 042127 | 531366  8146976 | Carretera Iquitos – Nauta, Provincia Maynas , Loreto |
| 07/11/2012 | *Piper sancti-felicis* Trel | Leaves and stems | 006367 | 0713020  9622125 | Estación Isula-Rio Napo, Lago Sunimiraño, Loreto |
| 14/12/2020 | *Piper stellipilum* (Miq.) C. DC | Leaves and stems | 039893 | 513250  8111253 | Estación Isula-Rio Napo, Lago Sunimiraño, Loreto |
| 6/12/2020 | *Piper trigonum* C. DC. | Leaves and stems | 23124 | 231733  8559990 | Ambito del valle de Kosñipata, distrito de Kosñipata, Provincia San Pedro, Chontachaca, Cusco |
| 6/12/2020 | *Piper verruculosum* C. DC. | Leaves and stems | 23122 | 215490  8544039 | Ámbito del valle de Kosñipata, Buenos Aires, Cusco |
| 29/12/2009 | *Piper xanthostachyum* C.DC. | Leaves | 10491 | 526941  8141988 | Alpahuayo Mishana, Carretera Iquitos - Nauta, Provincia Maynas, Loreto. |

**Supporting information 3.** Clustered Image Map (CIM) performed on the two-blocks of data sets (*in extenso*)


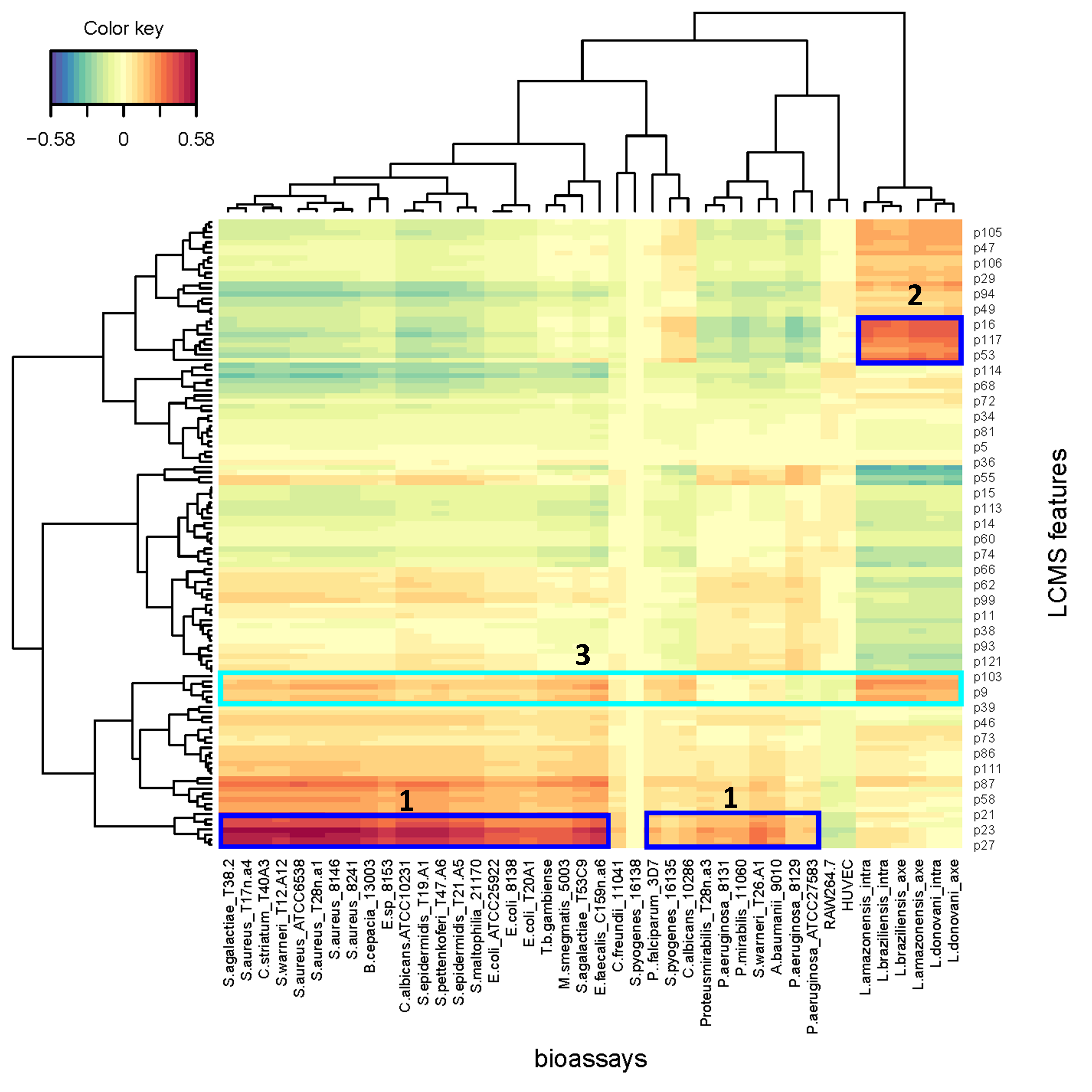

Supplement: Supplementary file 3 [file Table1.DOCX]
